# Comparative Cochlear–Vestibular Aging Reveals Age‐Aligned Mitochondrial Ultrastructural Burden, Mitophagy–Autophagy Remodeling, Synaptic Uncoupling, and Sensory Functional Decline

**DOI:** 10.1111/acel.70593

**Published:** 2026-06-23

**Authors:** Jingyi Xie, Xujia Zhang, Jinyi Tian, Yulu Yan, Ke Shi, Yongqi Pan, Yanni Zhang, Zichen Chen, Jianbin Sun, Hui Lv, Jingguo Chen, Xiaoyong Ren, Teru Kamogashira, Xiaotong Zhang, Ying Gao

**Affiliations:** ^1^ The Second Affiliated Hospital of Xi'an Jiaotong University Department of Otorhinolaryngology‐Head and Neck Surgery Xi'an China; ^2^ Shaanxi Provincial Key Laboratory for Precision Diagnosis and Treatment of Otorhinolaryngology Xi'an China; ^3^ Department of Otolaryngology and Head and Neck Surgery, Faculty of Medicine University of Tokyo Tokyo Japan

**Keywords:** age‐related sensory decline, cochlea–vestibular aging, mitochondrial ultrastructural injury, mitophagy–autophagy remodeling, synaptic uncoupling

## Abstract

Age‐related hearing loss and balance decline are prevalent features of organismal aging, yet how the cochlea and vestibular organs converge on shared cellular liabilities remains insufficiently resolved. In particular, whether mitochondrial ultrastructural injury and mitochondrial quality‐control programs co‐vary with synaptic vulnerability and sensory functional decline across these systems within an age‐resolved framework has not been clearly delineated. Here, we compared cochlear and vestibular aging in SAMP8 mice of different ages using integrated functional assays, region‐resolved quantification of hair cells and CtBP2/GluA2 synapses, cochlear NF200+ fiber area fraction, transmission electron microscopy, and targeted qPCR of mitophagy/autophagy–lysosome genes. The results show that ABR thresholds rose progressively across 5.6–32 kHz. VsEP exhibited age‐dependent threshold shifts and prolonged P‐wave latency. Relative to the magnitude of synaptic and functional changes, cochlear hair‐cell numbers were broadly preserved, although regional OHC loss was observed in middle‐to‐basal turns, whereas vestibular macular hair‐cell density declined with age. Ultrastructurally, the proportion of pathological mitochondria increased with age, featuring electron‐lucent matrix, disrupted cristae organization, and rounded/swollen profiles. What's more, guided by an adult‐versus‐aged transcriptomic screen nominating the Ca^2+^ extrusion gene Atp2b4, we derived z‐scored molecular indices, including a flux–burden signature (z(p62)–z(Lc3b)) and a TFEB–lysosome module. Descriptive coupling across age‐group means indicated that mitochondrial pathology burden aligned closely with high‐frequency ABR loss and basal synaptic uncoupling, and tracked the flux–burden signature more consistently than the TFEB–lysosome module. Together, these findings support age‐aligned associations among mitochondrial ultrastructural injury, molecular remodeling, synaptic vulnerability, and progressive sensory decline across cochlear and vestibular systems.

## Graphical abstract

This study maps age‐aligned cochlear and vestibular decline in SAMP8 mice, linking functional impairment with synaptic vulnerability, mitochondrial ultrastructural injury, and autophagy/mitophagy–lysosome transcriptional remodeling. The findings provide an integrated framework for understanding shared inner‐ear aging trajectories.

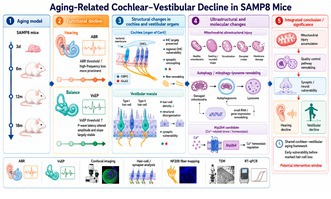

## 1 Introduction

Hearing and balance are foundational to healthy aging: when either system fails, communication, mobility, and independence decline. The global scale is substantial—nearly 2.45 billion people are projected to live with some degree of hearing loss by 2050, underscoring the urgency of understanding mechanisms that drive sensory aging and identifying tractable targets for intervention (Global Burden of Disease Study 2019 2021; Li et al. 2025).

Age‐related hearing loss (ARHL) and age‐related vestibular loss often co‐occur and jointly magnify disability risk, including reduced social participation, impaired daily functioning, and vulnerability to falls (Paplou et al. 2021; Coto et al. 2021; Politi et al. 2022; Aging Biomarker Consortium, Fu, Wang, et al. 2025). While the cochlea and vestibular organs share embryologic origin and rely on hair‐cell–afferent synapses to encode sensory information, they are typically investigated as separate systems (Paplou et al. 2021). In the cochlea, a substantial body of work has highlighted the importance of synaptic degeneration (synaptopathy)—loss or dysfunction of ribbon synapses between inner hair cells and spiral ganglion neurons—as a potentially early and functionally meaningful substrate of auditory aging (Xie et al. 2024; Liu et al. 2024). In parallel, vestibular aging is increasingly recognized as a multi‐level process involving peripheral end‐organs and central pathways, with documented links to balance dysfunction and broader neurological health (Coto et al. 2021; Politi et al. 2022). Across both systems, mitochondrial integrity is a plausible convergent liability (Xu and Yang 2025). Sensory hair cells and their synaptic machinery operate under high energetic demand and Ca^2+^ flux, conditions that can amplify susceptibility to mitochondrial dysfunction (Xu and Yang 2025). Reviews of sensorineural hearing loss emphasize mitochondrial involvement across etiologies and highlight unresolved challenges in translating mitochondrial targeting into durable clinical benefit (Xu and Yang 2025; Goman et al. 2025). Mitochondrial quality control—particularly mitophagy and its coordination with the autophagy–lysosome system—provides a mechanistic framework for how damaged organelles are recognized and cleared, with transcriptional programs (e.g., TFEB‐regulated lysosomal biogenesis) increasingly implicated in aging and stress adaptation (Yang and Wang 2021; Park et al. 2022; Curnock et al. 2023).

Despite these advances, several gaps limit mechanistic inference at the systems level. First, cross‐organ, age‐resolved comparisons that measure cochlear and vestibular function alongside matched cellular readouts remain scarce; as a result, it is difficult to determine whether these organs decline through shared or divergent trajectories, and which structural features best explain functional impairment (Paplou et al. 2021; Coto et al. 2021). Second, even within the cochlea, synaptopathy is often inferred from limited endpoints or studied in isolation, complicating efforts to connect synaptic vulnerability to broader cellular programs that govern resilience (Xie et al. 2024; Liu et al. 2024). Third, mitochondrial quality‐control pathways are frequently assessed at a single level (e.g., gene expression or ultrastructure), but integrated evidence linking mitochondrial ultrastructural burden to synaptic integrity and sensory function across the cochlea and vestibule is still limited (Paplou et al. 2021; Xu and Yang 2025; Yang and Wang 2021).

Finally, accelerated‐aging models such as SAMP8 provide an opportunity to capture age‐dependent sensory changes on an experimentally tractable timescale, yet many studies emphasize auditory thresholds without simultaneously mapping vestibular physiology and mechanistic tissue signatures (Goman et al. 2025; Menardo et al. 2012).

To address these gaps, we designed an age‐resolved, cross‐organ study in SAMP8 mice that integrates (i) auditory and vestibular physiology, (ii) region‐resolved synaptic and cellular quantification, (iii) mitochondrial ultrastructure, and (iv) targeted transcriptional profiling of mitophagy/autophagy–lysosome programs. Our goal was to determine whether age‐dependent mitochondrial injury and remodeling of mitochondrial quality‐control‐related signatures co‐vary with synaptic vulnerability and functional decline, and whether comparing cochlear and vestibular readouts within a unified framework would reveal organ‐ and region‐specific patterns relevant to sensory aging. To further anchor candidate pathway selection in unbiased biology, we incorporated an adult‐versus‐aged transcriptomic screen that nominated upstream cellular stress signatures (including Ca^2+^ handling) to guide targeted downstream quantification.

In the sections that follow, we first describe the animal cohorts and the integrated experimental pipeline. We then present age‐resolved auditory and vestibular functional phenotyping, followed by region‐specific analyses of hair cells, synaptic integrity, and cochlear innervation. We next quantify age‐associated mitochondrial ultrastructural pathology and evaluate targeted mitophagy/autophagy–lysosome transcriptional modules informed by transcriptomic nomination. Finally, we synthesize these cross‐scale endpoints to describe age‐aligned trajectories of cochlear and vestibular aging.

## 2 Materials and Methods

### 2.1 Animals, Ethics Approval, and Housing

Senescence‐accelerated mouse prone 8 (SAMP8) mice were used. Male and female mice were used in approximately equal proportions across groups; data were pooled because separate sex‐stratified analysis was precluded by sample size; no overt sex differences were noted in preliminary inspections. The present study was approved by the Medical Ethics Committee of Xi'an Jiaotong University (Approval No. 2021‐847), and all procedures were conducted in accordance with relevant guidelines and regulations. All mice were obtained from the Laboratory Animal Center, Xi'an Jiaotong University Health Science Center, weighed prior to experiments, and housed under standard conditions with free access to food and water. Mice were studied at four ages: postnatal Day 3 (3d), 6 months (6 m), 12 months (12 m), and 18 months (18 m). We note that the 3d group represents a developmental stage rather than a young‐adult baseline; accordingly, comparisons spanning 3d to adulthood were interpreted as developmental‐to‐aging trajectories, whereas adult aging inferences were based primarily on comparisons among the 6 m, 12 m, and 18 m groups.

### 2.2 Anesthesia, Perfusion, and Tissue Collection

Mice were anesthetized with xylazine (20 mg/kg) and ketamine (100 mg/kg). Auditory brainstem response (ABR) testing was performed first and vestibular sensory‐evoked potential (VsEP) testing immediately thereafter within the same anesthetic session. After physiological testing, animals were euthanized by decapitation, and cochlea/vestibular tissues were harvested for downstream applications. For immunofluorescence experiments, mice were transcardially perfused with 20 mL normal saline followed by 20 mL 4% paraformaldehyde (PFA). After decapitation, cochleae were dissected and immersed in 4% PFA at 4°C overnight, washed in PBS (3×), and decalcified in 10% EDTA on a shaker at room temperature for 3–5 days. For TEM, 3d mice were cryo‐anesthetized on ice, decapitated, and cochleae were immediately immersed in electron microscopy fixative (see below). Adult TEM specimens were collected following perfusion fixation as detailed in the TEM section.

### 2.3 Auditory Brainstem Responses (ABR)

ABRs were recorded using a TDT auditory function testing system (USA) in a sound‐attenuated chamber. Following anesthesia, mice were placed on a thermostatically controlled DC heating pad to maintain body temperature. ABR recordings were obtained using three subcutaneous electrodes: recording at the vertex, reference near the mastoid, and ground on the dorsal surface of the buttocks. Pure tone stimuli were delivered via a speaker positioned 3–5 cm from the tested ear. Stimulus frequencies were 5.6, 8, 16, 24, and 32 kHz, with a maximum intensity of 90 dB SPL.

Intensity was reduced in 10 dB SPL steps and subsequently in 5 dB SPL steps as threshold was approached. If ABR waveforms were inconsistent between two consecutive trials, intensity was increased in 5 dB increments until reproducible waveforms were obtained; this intensity was recorded as the ABR threshold. The same procedure was applied to the right ear. Each stimulus intensity was repeated 500 times per session and presented at least twice, with reproducible waveforms required. Thresholds were determined by manual waveform inspection by an experimenter blinded to age group, with reproducibility across repeated runs required. For bilateral recordings, left and right thresholds were averaged to generate a single animal‐level outcome for statistical analysis. For high‐frequency sensitivity, a composite threshold was calculated as HF_avg_24_32 = mean (24, 32 kHz).

### 2.4 Vestibular Sensory‐Evoked Potentials (VsEP)

VsEPs were recorded immediately after ABR measurements. The setup included an acceleration stimulus delivery system and an evoked potential recording system. Under anesthesia, mice were placed prone and their heads immobilized using a head fixation device (Model SG‐4 N, Narishige, Japan). The anterior incisors were secured to a maxillary fixation ring, and bilateral rotational screws stabilized the head anterior to the ears.

The head fixation system was connected to a vibration generator (Model S‐0105, Asahi Manufacturing, Japan) generating two stimulus waveforms: symmetric parabolic ramp (SPR; triangular) and linear acceleration ramp (SPLR; square). Acceleration (Model 352C65) and pressure (Model P51) transducers (PCB Piezotronics, USA) were used for detection and calibration, ensuring the VsEP peak jerk was calibrated to 0 dB re. 1 g/ms. The effective stimulus waveform typically had a 1 ms onset followed by a 2 ms peak jerk.

Stimulus intensity was initially set to 14.5 dB re. 1 g/ms and decreased in 10 dB steps. If no stable waveform was evoked, intensity was increased in 5 dB increments until a reproducible response was observed. Under each vibration mode, each intensity was repeated 512 times per session and presented at least twice with reproducible waveforms required; intensities were alternated to minimize habituation. VsEP outcomes included threshold, P‐wave latency, P‐wave amplitude, and the slope parameter K, defined as P‐wave slope (amplitude/rise time). Thresholds were determined by manual assessment of waveform reproducibility by an experimenter blinded to age group, using the criteria described above. Unless otherwise specified, VsEP outcomes were analyzed at the animal level.

### 2.5 Immunofluorescence Staining

After fixation by using 4% PFA (Beyotime) and (for adult cochleae) EDTA decalcification, tissues were permeabilized and incubated in antibody solution containing 3% Triton X‐100 and 5% BSA in PBS. Primary antibodies were typically used at 1:200 and incubated overnight at 4°C, followed by PBS washes (5 min × 3). Secondary antibodies were typically used at 1:500 and incubated for 1 h at room temperature in the dark, followed by PBS washes (5 min × 3). Samples were mounted with VECTASHIELD and kept at 4°C overnight before imaging. Hair cells were visualized using phalloidin, nuclei were counterstained with DAPI, and supporting cells were labeled with SOX2 where indicated. Synapses were labeled with CtBP2 and GluA2. NF200 was used to visualize cochlear fibers; TUJ1 signal was primarily interpretable in vestibular maculae in our preparations. Primary antibodies: SOX2 (mouse monoclonal, ab79351, Abcam), CtBP2 (mouse IgG1, 612044, BD Biosciences), GluA2/GluR2 (mouse IgG2a, MAB397, Chemicon), TUJ1 (mouse IgG2a, MMS435P, Covance), NF200 (rabbit, N4142, Sigma). Secondary antibodies: goat anti‐mouse IgG (Alexa Fluor 647, ab150115, Abcam), goat anti‐mouse IgG1 (Alexa Fluor 488, A21121, Thermo Fisher), rat anti‐mouse IgG2a (Alexa Fluor 647, ab172325, Abcam), goat anti‐rabbit IgG (Alexa Fluor 405, A‐31556, Thermo Fisher). Additional reagents included CoraLite Plus 488–conjugated phalloidin and VECTASHIELD mounting media (H‐1000; H‐1200 with DAPI).

Confocal imaging was performed on a Nikon A1 microscope using 20× and 60× objectives. Z‐stacks were acquired with step sizes of 2 μm for hair‐cell imaging and 1.5 μm for synapse and nerve fiber imaging. Images were acquired/managed using NIS‐Elements C‐ER and processed/quantified using Fiji (ImageJ), NIS‐Elements C‐ER, and NIS‐Elements Viewer.

### 2.6 Quantification of Hair Cells, Synapses, and Cochlear Fibers

All imaging‐based quantifications were summarized at the specimen level by averaging across fields of view. For each specimen, three random fields were analyzed per region or zone, and field‐level measurements were averaged to generate a specimen‐level estimate used for statistical analysis. Specifically, for cochlear whole mounts, the organ of Corti was analyzed in the apical, middle, and basal turns. For each turn, three random fields of view were imaged and counted; the three fields were averaged to yield a turn‐level estimate per cochlea. For vestibular maculae, central and marginal zones were quantified separately using three random fields per zone per specimen. Synaptic puncta were quantified from CtBP2 (presynaptic ribbon) and GluA2 (postsynaptic puncta). Paired synapses were defined in Fiji using a centroid proximity threshold of < 0.4 μm between CtBP2 and GluA2 puncta. The same image‐processing and puncta‐detection thresholds were applied within each marker set and acquisition batch. To improve reproducibility, 10% of images were manually verified. To minimize confounding from variable hair‐cell numbers across images, cochlear synapse metrics were normalized to fixed hair‐cell counts: IHC‐related synapse counts were normalized per 30 IHCs, and OHC‐related synapse counts were normalized per 90 OHCs. For cochlear analyses, synapse density and paired/unpaired proportions were quantified for each turn. For vestibular maculae, synapse density and paired proportion were quantified separately in the central and marginal zones. Cochlear innervation was quantified as NF200+ fiber area fraction within standardized regions of interest in apical, middle, and basal turns. For each turn, three random fields of view were analyzed and averaged to generate a turn‐level estimate per cochlea.

### 2.7 Transmission Electron Microscopy (TEM) and Pathological Mitochondria Scoring

For TEM, three mice were randomly selected from each age group; each mouse was provided with one cochlea, with no restriction on the left or right side. The EM fixative was prepared by mixing 20 mL of 2.5% glutaraldehyde, 80 mL of formalin, and 100 mL of sterile PBS. For 3d mice, cochleae were collected immediately after decapitation and immersed directly in EM fixative. Adult mice were transcardially perfused with 20 mL sterile PBS followed by 20 mL fixative. After decapitation, temporal bones were excised, auditory bullae were opened, and ossicles were removed. Samples were post‐fixed in 2.5% glutaraldehyde at 4°C for 48 h, washed three times with sterile PBS, and decalcified in 0.12 M EDTA (pH 7.2) at 4°C for 1 week. Tissues were washed, dehydrated through graded ethanol, and embedded in epoxy resin. Semi‐thin sections (2 μm) were stained with toluidine blue for orientation, and regions of the cochlear basilar membrane and vestibular saccule were selected for ultrathin sectioning. Ultrathin sections were stained with uranyl acetate and lead citrate and examined on a Hitachi H9500 transmission electron microscope.

For quantitative scoring, three random fields of view were analyzed per cochlea, with 10–30 mitochondria per field. Scoring was performed by a single experimenter blinded to age group. Each mitochondrion was classified as pathological (0/1) if any of the following features were present: swelling or rounding, vacuolization, cristae disruption (fracture, sparsity, or disorganization), or outer membrane rupture. The primary TEM endpoint was the percentage of pathological mitochondria per cochlea, calculated by averaging the pathological fraction across the three sampled fields; thus, the cochlea/specimen rather than the individual mitochondrion served as the statistical unit.

### 2.8 Transcriptomic Nomination (Adult vs. Aged) and Functional Grouping

Publicly available adult‐versus‐aged cochlear transcriptomic data were retrieved from the Gene Expression Omnibus (GEO) and re‐analyzed to nominate aging‐associated candidates and inform subsequent targeted profiling. Raw FASTQ files were processed using Cell Ranger (v9.0.1) to generate gene–barcode count matrices, and downstream analyses were performed in Python (v3.12). After quality control, samples were merged and batch effects were corrected prior to clustering and marker‐based annotation, yielding 24 cell types; hair cells were subsetted using canonical markers (Pou4f3 and Pcp4). Differential expression was assessed between adult and aged hair‐cell groups (as defined in this study), with genes meeting nominal *p* < 0.05 and |log2FC| > 0.25 considered differentially expressed (107 DEGs in total; 73 downregulated and 34 upregulated). To support biological interpretation and candidate prioritization, DEGs were analyzed for pathway and ontology enrichment using Enrichr (GO Biological Process/Cellular Component/Molecular Function 2021, KEGG 2019 Mouse, WikiPathways 2024 Mouse, MSigDB, and BioCarta), and enriched terms were further consolidated into study‐relevant functional themes, including Ca^2+^ homeostasis, mitochondrial pathways, lysosome biology, autophagy/mitophagy, and synapse/axon guidance. Because increased variability reduced statistical significance for subsets of genes in volcano plots, genes were additionally ranked by log2FC and subjected to gene set enrichment analysis (GSEA); a mitochondria‐related gene set was curated from GeneCards using the keyword “mitochondrial,” supporting coordinated downregulation of mitochondrial metabolic program in the aged group. Among the nominated candidates, the Ca^2+^ extrusion gene Atp2b4 was identified as age‐upregulated (log2FC = 1.62; *p* = 0.044) and was prioritized for downstream targeted profiling.

### 2.9 RT–qPCR

Each qPCR value represents one biological specimen (cochlear tissue sample) and was analyzed at the specimen level. Primers were designed and synthesized by Tsingke Biotechnology (Beijing, China). qRT‐PCR was performed using TB Green Premix Ex Taq II (Takara). Reaction mixtures contained TB Green Premix Ex Taq II (Tli RNaseH Plus), ROX Reference Dye II (50×), cDNA template, gene‐specific primers, and deionized water. Amplification conditions were: 95°C for 30 s, followed by 40 cycles of 95°C for 5 s and 60°C for 30 s. Melting curve analysis confirmed product specificity. Primer sequences are provided in Table 1. Gene expression was normalized to β‐actin and Gapdh, and relative quantification was calculated using the 2^−ΔΔCt method. Fold changes were log2‐transformed for visualization and analysis.

**TABLE 1:** List of primer sequences for target genes.

Gene	Forward primer	Reverse primer
Pink1	CCTTTCAACAGCTCCAGTGTAGA	GGCAAGCCCAAAGATTTCATAGG
Parkin	CCGAATCACCTGACGGTTCA	TGGGTTTAACTGCTGGACCT
Atg4a	GTTCAAGACAAATGGGCAGGAAA	CAATTCCACCGGCAACTTGACTA
Lc3b	CTGTAAGGAGGTGCAGCAGATC	ATGATCTTGACCAACTCGCTCAT
P62	CACAGGCACAGAAGACAAGAGTA	CCTGTAGATGGGTCCACTTCTTT
Ctsd	TGCCTCTTATCCAGGGTGAGTAT	ATTGTCTTTCCACCCTGCGATAC
Atp2b4	GCAGTGGATGTGGTGTCTCTTTA	GTATCCCTGCTGATGTCCTCTTT
Mcoln3	TGTGTGCCAGGTCTGTGATTAG	TGTGAGACTCTTGGCTTGGATTT
Becn‐1	GAGGTACCGACTTGTTCCCTATG	GGTCAAACTTGTTGTCCCAGAAA
Tfeb	GCATCAGAAGGTTCGGGAGTATC	CAGGCGCATAATGTTGTCAATGA
Bnip3	GCATGAGAAACACAAGCGTTATG	GTCAGACGCCTTCCAATGTAGAT
Bnip3l	CTTCCTCGTCTTCCATCCACAAT	ACATGATCTGCCCATCTTCTTGT
Dnm1l	AGCAATTACAGCACACAGGAATT	TACGCTAGCTCAATTGCCACTAA
Fundc1	AATCGAGTATTTGGCCACAGTTC	GTGACTGGCAACCTGAAGAAGAA
Mtor	TTGTTGCCTCCGATTGTGAAATT	GAGGCGTAGTCAGTGAAGTCTAG
Prkaa1	GTCAAAGCCGACCCAATGATATC	AGTCCCTGATTTGGCTTCTGTAA
Ulk1	CAAACACTGCTGGGAAAGGAAAT	ACTCCATGACCAGGTAGACAGAA
Tsc1	TGAAGAGCTGTGCAAACCTTTAG	ATGTGCAATACCGGCTGAGAATT
β‐Actin	GCTGTGCTATGTTGCTCTAGACT	GTGTTGGCATAGAGGTCTTTACG
Gapdh	TGTCAAGCTCATTTCCTGGTATG	GGGATGGAAATTGTGAGGGAGAT

### 2.10 Z‐Score Module Indices

To construct composite molecular indices, log2 fold changes were z‐scored across all samples: z = (x − mean)/SD. Indices used for integrative analyses included: (i) Flux–burden index: z(p62) − z(Lc3b); (ii) TFEB–lysosome module score: mean[z(Tfeb), z(Ctsd), z(Mcoln3)]. These indices were used as descriptive transcriptional summaries for integrative analyses and should not be interpreted as direct measurements of autophagic flux, mitophagy activity, or lysosomal function.

### 2.11 Statistical Analysis

Statistical analyses were performed using GraphPad Prism 10 for macOS (Version 10.3.0 (461), July 26, 2024). For outcomes assessed across age and region (apical/middle/basal; central/marginal), two‐way ANOVA was used with multiple‐comparison correction (e.g., Sidak) as implemented in Prism. Data are presented as mean ± standard deviation (SD). A *p*‐value of less than 0.05 was considered statistically significant. Significance is coded as *p* < 0.05 (*), < 0.01 (**), < 0.001 (***), < 0.0001 (****). Unless otherwise specified, plots show means with SD error bars. For physiological assays (ABR and VsEP), the animal served as the statistical unit. For imaging‐based assays, field‐level measurements were first averaged within each specimen, and specimen‐level values were used for group comparisons. For TEM, pathological fractions were first calculated at the field level and then averaged per cochlear specimen. A summary of sample sizes, aggregation levels, and statistical units for each assay is provided in Table S1.

Linear regression was used for descriptive trajectory‐alignment plots. When individual‐level pairing across modalities was incomplete, integrative coupling plots were generated using age‐group means (3d, 6 m, 12 m, 18 m; *n* = 4 age points). Simple linear fits with 95% confidence bands were used for visualization, and *R* ^2^ was reported as a descriptive index of trajectory alignment; inferential correlation testing was not emphasized due to the limited number of age points and the use of group means rather than paired individual measurements.

## 3 Results

### 3.1 Age‐Dependent Sensory Functional Decline in Auditory and Vestibular Systems

#### 3.1.1 ABR Thresholds Demonstrated a Progressive Increase With Advancing Age

**FIGURE 1:**
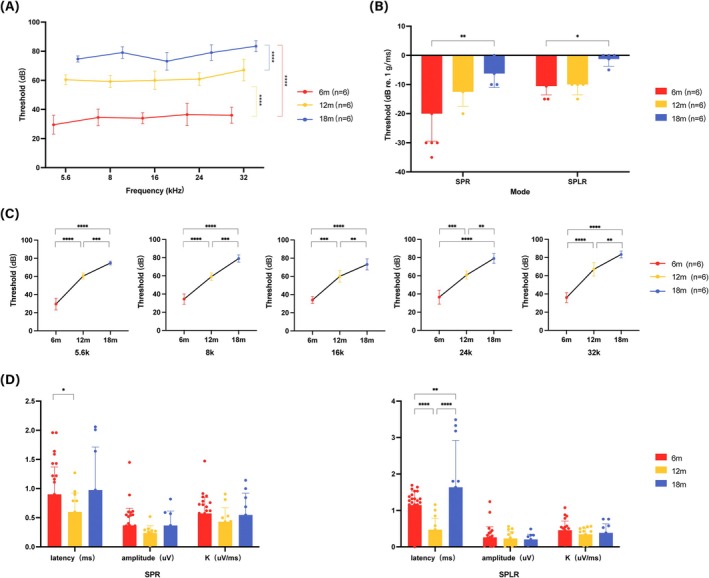
Age‐dependent decline in auditory and vestibular function. (A) Time‐course of mean ABR thresholds at multiple frequencies across age groups. (B) VsEP thresholds under SPR and SPLR conditions across age groups. (C) Temporal variations in frequency‐specific ABR threshold differences among groups. (D) Comparative analysis of VsEP P‐wave latency, amplitude, and slope (K) across age groups in SPR and SPLR modes. Unless otherwise specified, plots show means with SD error bars; n represents animals per group. Significance is coded as *p* < 0.05 (*), < 0.01 (**), < 0.001 (***), < 0.0001 (****).

Auditory Brainstem Response (ABR) waveforms were successfully recorded in all mice. With advancing age, the ABR thresholds exhibited a significant progressive increase across all tested frequencies, indicating a gradual decline in auditory sensitivity (*p* < 0.05; Figure 1A,C). Specifically, this age‐related threshold elevation was statistically significant at specific frequencies among 5.6, 8, 16, 24, and 32 kHz (*p* < 0.05; Figure 1C). Notably, the impairment was more prominent in the high‐frequency range (24–32 kHz), further suggesting that age‐related auditory decline preferentially affects the broader frequency region encompassing high frequencies.

#### 3.1.2 The VsEP Threshold and P‐Wave Latency Were Susceptible to Aging, Whereas the Wave Amplitude and Slope Remained Relatively Preserved

Vestibular sensory evoked potential (VsEP) measurements indicated an age‐related decline in vestibular function. Under both SPR and SPLR stimulation paradigms, VsEP thresholds showed an overall upward shift with age. Statistically significant differences were observed between the 6‐ and 12‐month age groups under both paradigms (*p* < 0.05; Figure 1B), suggesting a reduction in vestibular sensitivity that became more pronounced in later stages of aging. Beyond threshold shifts, we further analyzed the latency, amplitude, and slope of the P‐wave across age groups under different paradigms. The P‐wave latency exhibited age‐dependent alterations, demonstrating a biphasic trend (an initial shortening followed by a prolongation) in both paradigms. Under the SPR paradigm, a statistically significant difference in latency was found between the 6‐ and 12‐month groups (*p* < 0.05; Figure 1D). The age‐related changes were more pronounced under the SPLR paradigm, with significant differences observed among all three age groups (*p* < 0.05; Figure 1D). These findings suggest that aging primarily affects the temporal characteristics (e.g., conduction velocity/synchronization) of the vestibular response. In contrast, both the P‐wave amplitude and the slope parameter K showed minimal overall variation across age groups and did not exhibit a consistent, statistically significant age‐related declining trend.

### 3.2 Differential Vulnerability of Cochlear and Vestibular Sensory Epithelia During Aging

#### 3.2.1 Cochlear Sensory Epithelium: Selective Vulnerability Predominantly Affecting Outer Hair Cells, With Relative Preservation of Inner Hair Cells and SOX2‐Positive Supporting Cells in Adulthood

**FIGURE 2:**
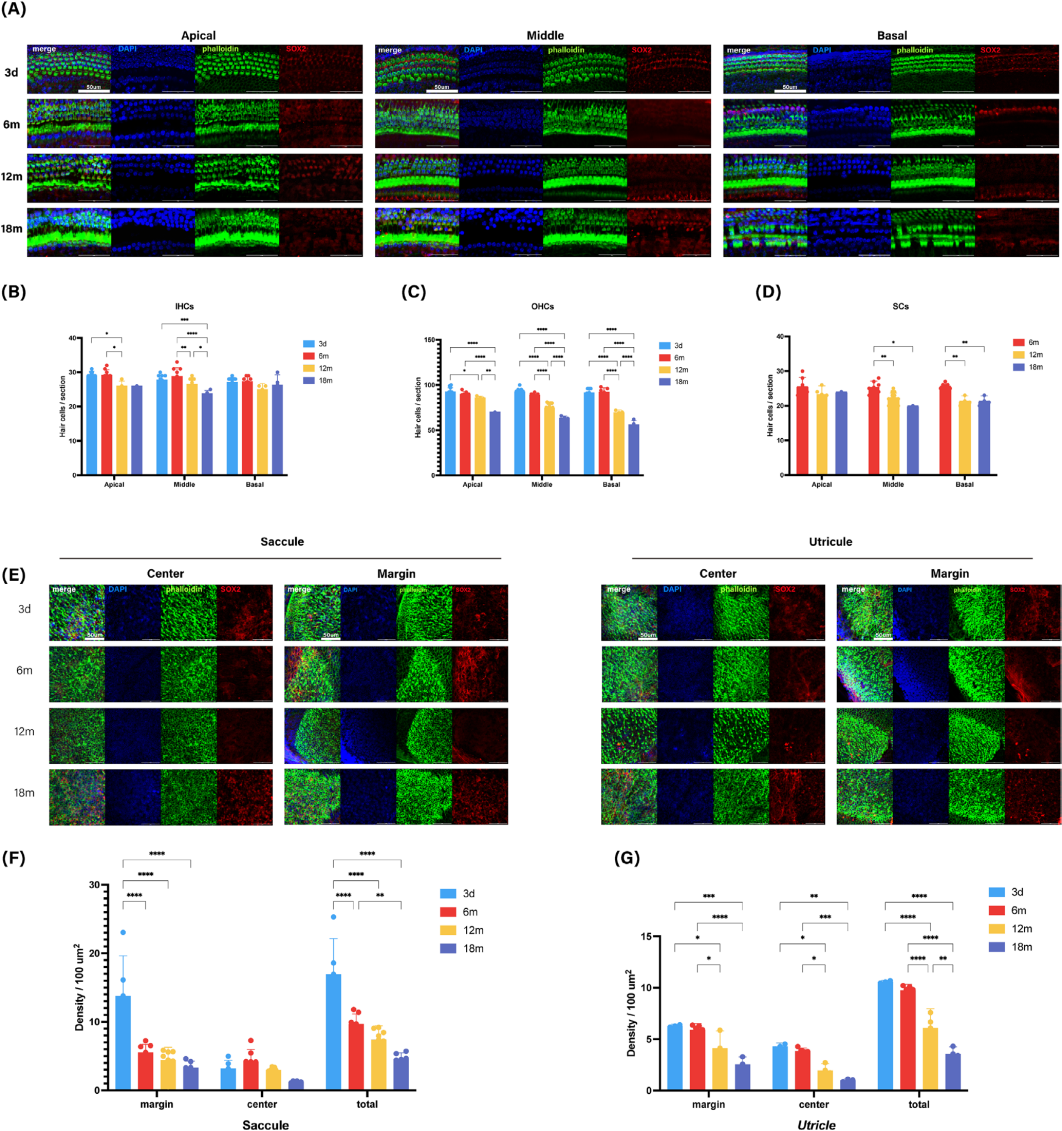
Differential vulnerability of cochlear and vestibular sensory epithelia during aging. (A) Representative cochlear whole‐mount images from apical, middle, and basal turns in young and aged mice (as indicated). (B–D) Quantification of inner hair cells (IHCs), outer hair cells (OHCs), and Sox2+ supporting cells across cochlear turns at each age group. (E) Representative images of the saccular and the utricular macula showing marginal and central zones in young and aged mice. (F) Hair cell density in the saccular and the utricular macula (cells per 100 μm^2^) quantified separately in marginal and central zones, with total density shown. (G) Representative images of the utricular macula showing marginal and central zones in young and aged mice. Whole‐mount preparations of the cochlea and otolith organs were stained with phalloidin (F‐Actin of stereocilia bundles), DAPI (nuclei), and Sox2 (supporting cells). Unless otherwise specified, plots show means with SD error bars; sample sizes and statistical units are summarized in Table S1. Significance is coded as *p* < 0.05 (*), < 0.01 (**), < 0.001 (***), < 0.0001 (****).

Whole‐mount cochlear quantification revealed age‐related selective remodeling, primarily involving outer hair cells (OHCs). The number of inner hair cells (IHCs) remained largely stable across the examined age series, with only a mild decline observed in specific cochlear turns of the oldest individuals. In contrast, OHCs exhibited a significant and progressive loss with age, which was particularly concentrated in the middle‐to‐basal turns (*p* < 0.05; Figure 2A–C). Notably, the basilar membrane of neonatal (3d) mice exhibited widespread SOX2 staining (Figure 2A), potentially indicating incomplete supporting cell differentiation. Therefore, supporting cell quantification was restricted to the 6‐, 12‐, and 18‐month groups to avoid confounding developmental effects. Within the 6–18 month series, SOX2‐positive supporting cell counts were generally maintained, showing only mild age‐related variations in specific turns. A statistically significant loss was found specifically between the 6‐ and 12‐month groups in the middle and basal turns (*p* < 0.05; Figure 2D). In summary, cochlear aging in SAMP8 mice was characterized by comparatively preserved IHC numbers and supporting‐cell labeling during adulthood, together with region‐selective OHC vulnerability, rather than by widespread cochlear hair‐cell depletion.

#### 3.2.2 Vestibular Otolithic Maculae: Generalized Hair Cell Density Decline Accompanied by Central SOX2+ Cell Accumulation and Hair Cell Disorganization

Analysis of the vestibular otolith organs revealed that, unlike the cochlea, the maculae (saccule and utricle) exhibited age‐related hair cell density declines in both the peripheral and central zones, leading to a significant reduction in overall macular hair cell density in aged mice (*p* < 0.05; Figure 2F,G; Figure S1). In addition to the quantitative loss, morphological abnormalities in hair cells were observed, including enlarged intercellular gaps, disoriented orientation, and focal depletion (Figure 2E), indicative of worsening structural disorganization. Notably, a progressively more prominent accumulation of SOX2‐positive cells was observed in the central zone of the maculae with advancing age (Figure 2E). This central increase in SOX2+ cells showed a temporal correlation with both the disorganization and density decline of hair cells. Compared to the pattern of preferential OHC vulnerability in the cochlea, the vestibular maculae displayed more widespread age‐associated epithelial remodeling, including hair‐cell loss and structural disorganization, which were more pronounced in the utricle. These changes occurred concomitantly with an altered regional distribution of the supporting cell marker.

### 3.3 Age‐Related Alterations in Synaptic Structures: Cochlea Exhibits Pre‐ to Post‐Synaptic Mismatch, While Vestibular System Shows Predominant Synaptic Density Decline

#### 3.3.1 Cochlea: Increased Pre‐/Post‐Synaptic Mismatch and Reduced Colocalization in IHC and OHC Regions Across Turns

**FIGURE 3:**
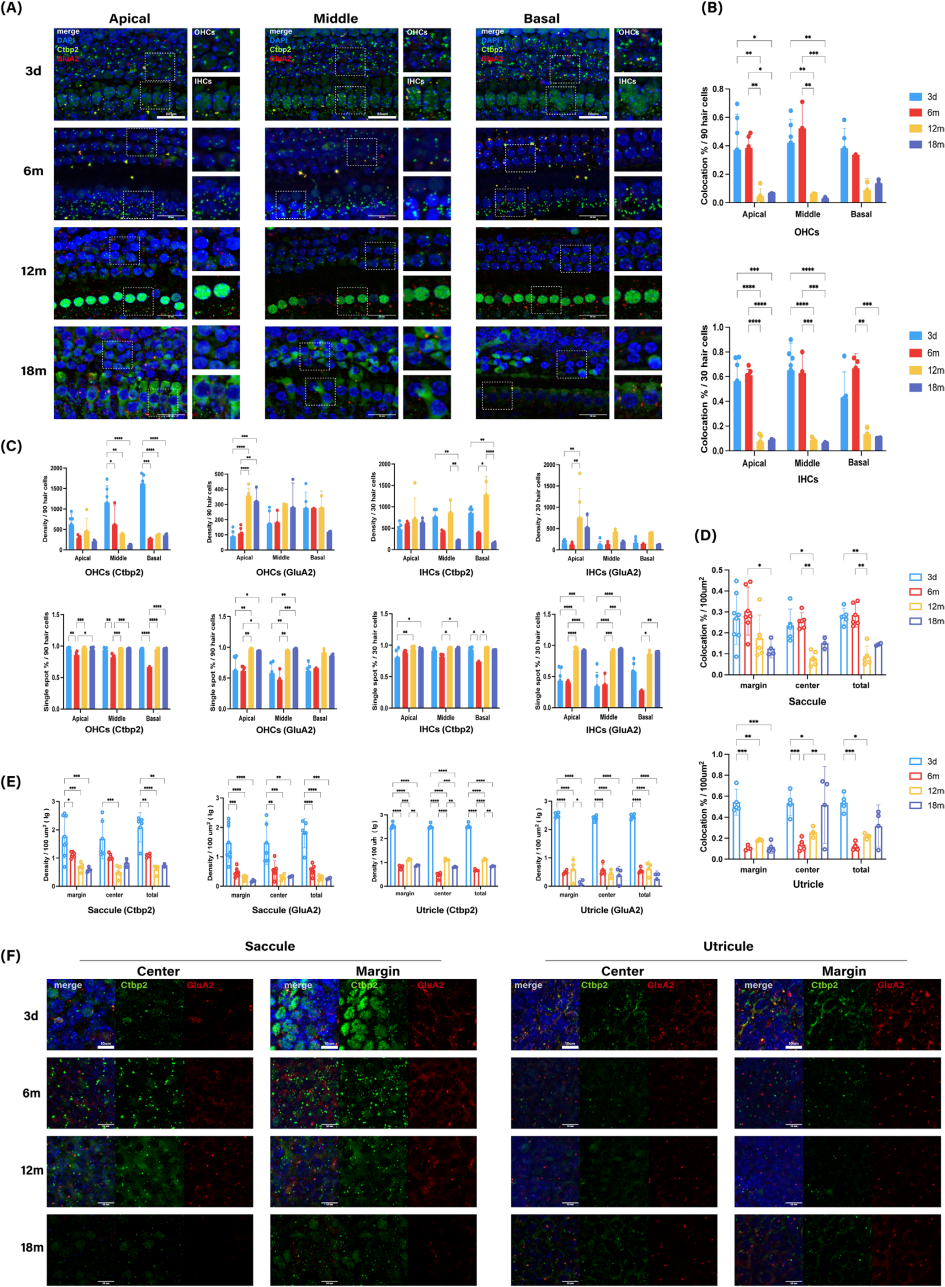
Age‐dependent synaptic uncoupling in the cochlea and vestibular maculae. (A) Representative cochlear images illustrating CtBP2 and GluA2 puncta in IHC and OHC regions across cochlear turns (apical, middle, basal; young vs. aged as indicated). (B) Percentage of matched (colocalized) CtBP2–GluA2 puncta in IHCs and OHCs across cochlear turns. (C) Density and percentage of single (unpaired) puncta (CtBP2‐only or GluA2‐only, as indicated) across cochlear turns. (D) Percentage of matched puncta (colocalization %) in saccule and utricle across ages. (E) CtBP2, GluA2, and matched synapse densities in the saccule and utricle, quantified in marginal and central zones, with total values shown. Synaptic densities in the cochlea were normalized to a fixed number of hair cells (OHC: Per 90 OHCs; IHC: Per 30 IHCs). (F) Representative vestibular macular images showing CtBP2 and GluA2 puncta in marginal and central zones (young vs. aged). Vestibular synaptic densities are presented per 100 μm^2^. Unless otherwise specified, plots show means with SD error bars; sample sizes and statistical units are summarized in Table S1. Significance is coded as *p* < 0.05 (*), < 0.01 (**), < 0.001 (***), < 0.0001 (****).

Cochlear synaptic structures in the IHC and OHC regions across different turns (apical, middle, basal) were quantitatively analyzed using double immunofluorescence labeling for CtBP2 (a presynaptic ribbon marker) and GluA2 (a postsynaptic AMPA receptor marker). The results revealed significant age‐related synaptic remodeling in the cochlea. The colocalization percentage between CtBP2 and GluA2 puncta showed an overall decreasing trend (Figure 3A), which was statistically significant (*p* < 0.05; Figure 3B). Conversely, the proportion of unpaired puncta (single spots, appearing as either CtBP2‐only or GluA2‐only signals) increased (Figure 3C). These findings indicate a progressive age‐associated reduction in the spatial alignment between presynaptic ribbons and postsynaptic receptor clusters, consistent with increasing pre‐ to post‐synaptic mismatch or uncoupling. These alterations were observable across multiple turns and were more pronounced in the basal turns (*p* < 0.05; Figure 3B,C), suggesting a spatially graded decline in synaptic connection stability during cochlear aging. Cochlear synaptic density was normalized to a fixed number of hair cells to account for the influence of hair cell loss.

#### 3.3.2 Vestibular System: Declined Synaptic Puncta Density and Reduced Colocalization in the Saccular and Utricular Maculae, Affecting Both Peripheral and Central Zones

In the vestibular otolith organs (saccular and utricular maculae), synaptic analysis was similarly performed using CtBP2/GluA2 double immunofluorescence, with quantitative assessments conducted separately for the peripheral (margin) and central zones (Figure 3F). The results showed that the density of both CtBP2 and GluA2 puncta in the maculae exhibited declining trends with age in both zones, resulting in an overall reduction in total synaptic density (Figure 3E). Concurrently, the synaptic colocalization percentage decreased in aged groups (*p* < 0.05; Figure 3D), indicating that the structural matching between pre‐ and post‐synaptic elements in the vestibular maculae is also compromised with age.

### 3.4 Age‐Related Decline in Cochlear Type II Afferent Fiber Integrity by Turn; Tuj1 Staining Provides Qualitative Observations

**FIGURE 4:**
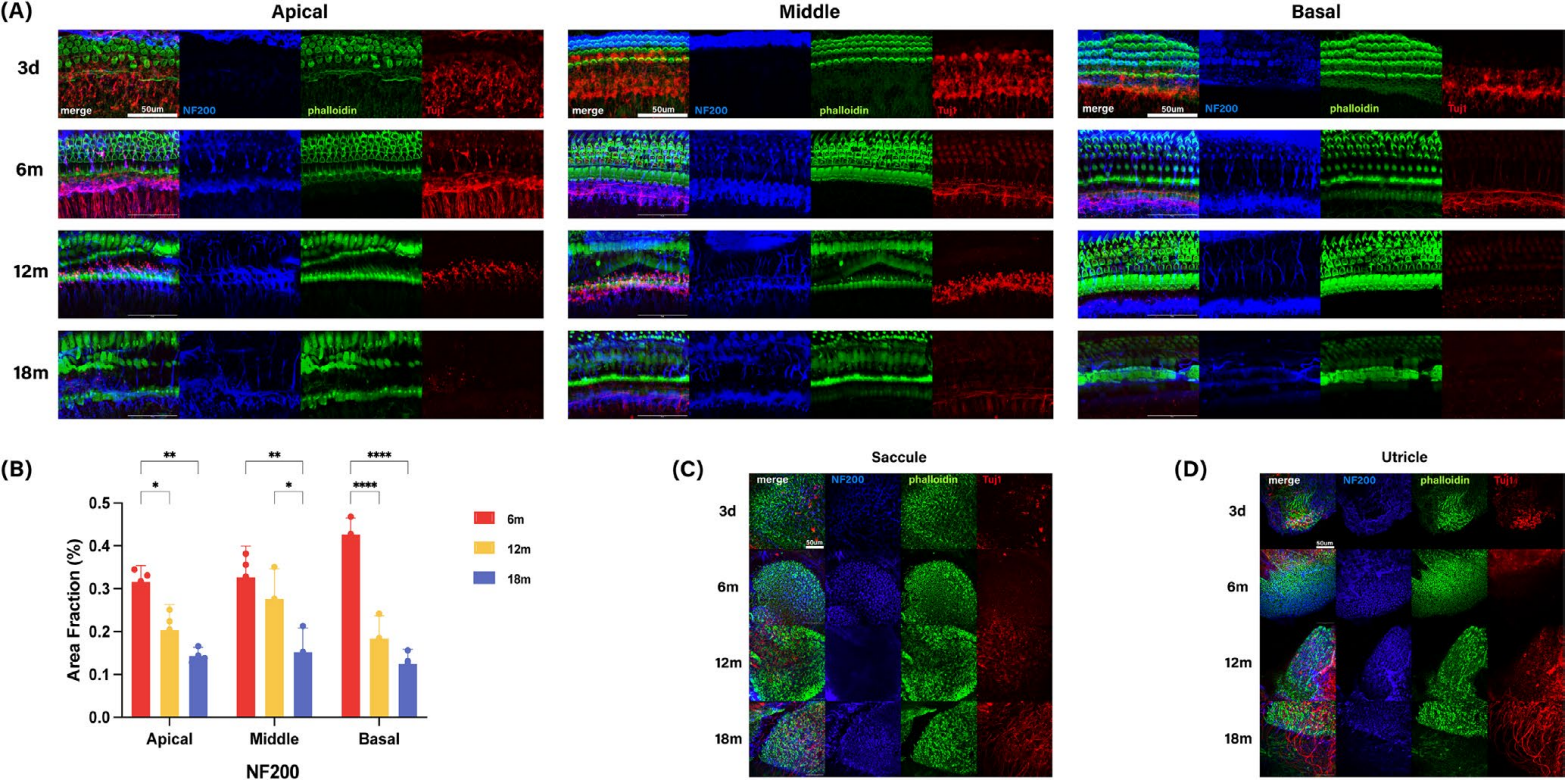
Turn‐resolved reduction of NF200^+^ cochlear fibers with age and qualitative remodeling of vestibular fibers (TUJ1). (A) Representative maximum‐intensity projection confocal images of cochlear whole mounts at 6, 12 and 18 months. Tuj1 (βIII‐tubulin) was included in the staining panel but is shown here for morphological reference only (see main text). (B) Quantification of NF200 area fraction by turn and age (mean ± SD). Area fraction was defined as (NF200‐positive pixels within ROI) / (ROI total pixels). (C–D) Representative confocal images of vestibular maculae (saccule and utricle, respectively). Unless otherwise specified, plots show means with SD error bars, sample sizes and statistical units are summarized in Table S1. Significance is coded as *p* < 0.05 (*), < 0.01 (**), < 0.0001 (****).

Analysis of cochlear turn revealed a progressive, age‐related decline in the NF200^+^ area fraction. Significant decreases were observed to varying degrees in the apical, middle, and basal turns, with the 18‐month group showing the lowest values (*p* < 0.05; Figure 4A,B). These findings suggest that the peripheral nerve fibers labeled by NF200 undergo thinning and degeneration during aging, exhibiting spatial variation along the cochlear axis. The staining protocol also included Tuj1 (βIII‐tubulin). However, within the basilar membrane preparations, Tuj1‐positive fiber trajectories were not consistently continuous or uniform across age groups, rendering automated quantification based on area or pixel intensity unreliable. Therefore, Tuj1 signals were evaluated qualitatively only. Representative confocal images (Figure 4A) demonstrated an age‐related reduction in the density of Tuj1‐positive fluorescent fibers across cochlear turns. This qualitative trend corroborates the quantitative decline in the NF200 area fraction, serving as supportive morphological evidence.

For the vestibular maculae (saccule and utricle), quantitative analysis was not performed due to sample size limitations. Nonetheless, representative images (Figure 4C,D) indicated that Tuj1‐positive fibers were relatively concentrated in the central zone. With advancing age, these central fibers appeared more prominent, densely packed, and exhibited more tortuous trajectories, often concomitant with focal depletion and disorganization of the hair cell layer. Given the descriptive nature of these vestibular data, these should be interpreted as qualitative evidence of possible regional fiber remodeling during aging. However, this preliminary conclusion requires quantitative verification with larger sample sizes or optimized staining and imaging conditions.

### 3.5 Mitochondrial Ultrastructural Burden Accumulates With Age and Coincides With Transcriptomic Themes Implicating Neural Connectivity and Ca^2+^ Homeostasis

#### 3.5.1 TEM Reveals Progressive Accumulation of Ultrastructurally Abnormal Mitochondria in the Cochlea

**FIGURE 5:**
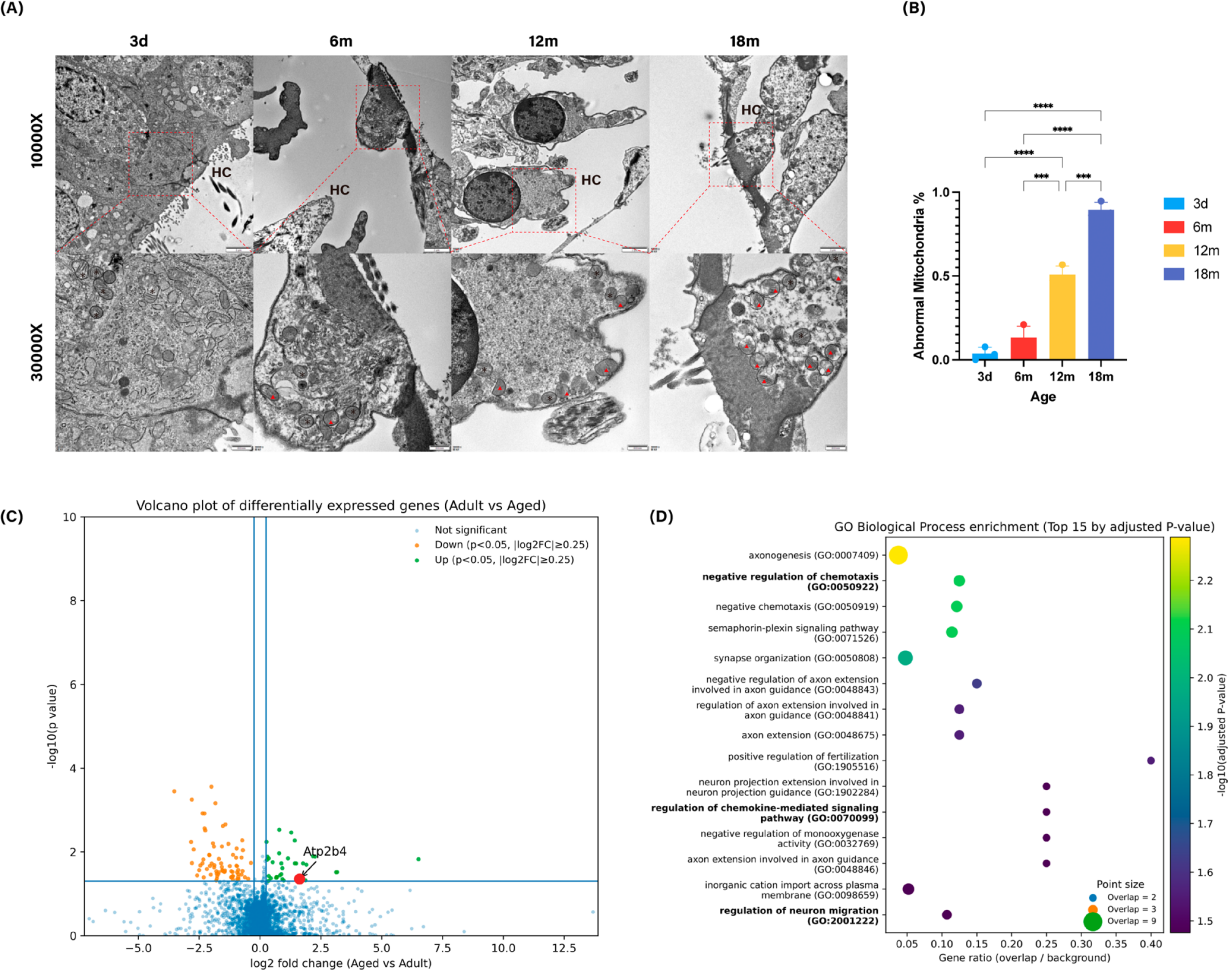
Age‐dependent accumulation of cochlear mitochondrial ultrastructural pathology and transcriptome‐guided nomination of candidate programs. (A) Representative transmission electron microscopy (TEM) images illustrating cochlear mitochondrial ultrastructure across the indicated ages. ✽ represents normal mitochondria, ▲ represents morphological changes in pathological mitochondria. (B) Quantification of the percentage of pathological mitochondria per cochlea. Each cochlea was analyzed from three randomly selected fields of view, with approximately 10–30 mitochondria scored per field. (C) Volcano plot of differential expression in hair cells comparing adult versus aged groups. *Atp2b4* is highlighted and labeled to indicate its nomination as a Ca^2+^‐handling candidate gene. (D) Gene Ontology Biological Process (GO BP) enrichment dot plot showing the top 10–15 terms ranked by adjusted *p* value. Dot size represents the number of genes mapped to each term, and dot color encodes the adjusted *p* value. Unless otherwise specified, plots show means with SD error bars; sample sizes and statistical units are summarized in Table S1. Significance is coded as *p* < 0.001 (***), < 0.0001 (****).

Transmission electron microscopy showed an age‐dependent increase in mitochondria with overt structural abnormalities, including rounded/swollen profiles, electron‐lucent matrix, vacuolization, loss of cristae organization progressing to cristae fragmentation/sparsity, and occasional outer membrane rupture (Figure 5A). Using the cochlea as the analytical unit (three random fields per cochlea; ~10–30 mitochondria per field), the fraction of pathological mitochondria increased stepwise with age (*n* = 3/group; *p* < 0.05; Figure 5B), supporting a descriptive pattern of age‐associated accumulation of mitochondrial ultrastructural abnormalities.

#### 3.5.2 An Adult‐Versus‐Aged Hair‐Cell Transcriptomic Screen Nominates Atp2b4 as a Ca^2+^‐Linked Candidate Anchor

The adult‐versus‐aged differential expression table contained 4567 genes. Under nominal criteria (*p* < 0.05 and |log2FC|> 0.25), 34 genes were upregulated and 73 downregulated. Atp2b4 was among nominally upregulated candidates (log2FC ≈ 1.624; *p* ≈ 0.044) but did not pass multiple‐testing correction (padj ≈ 0.675) (Figure 5C); accordingly, we treat Atp2b4 as a data‐driven nomination rather than a definitive age effect, motivating targeted downstream profiling aligned with Ca^2+^ stress and mitochondrial quality control.

#### 3.5.3 Functional Enrichment of Nominally Upregulated Genes Highlights Programs Related to Neurite/Synapse Organization and Ion/Ca^2+^ Handling

GO biological process enrichment of the nominally upregulated gene set highlighted neurite and synapse‐related programs (e.g., axonogenesis, neuron projection development, synapse organization), featuring genes such as NRP2, NRXN1, ROBO1, SEMA family members, MAP1B, PTPRD, and PPFIA2. In parallel, the “inorganic cation import across plasma membrane” term contained Atp2b4 together with Slc12a2 and Slc9a9, consistent with ion/Ca^2+^ homeostasis as a convergent theme (Figure 5D). Additional DEGs included stress‐response and trafficking‐related nodes (e.g., Gpx2/Cygb/Cystm1; Slc9a9/Dnase1), providing context for subsequent targeted assessment of mitochondrial quality‐control pathways (Figure S2).

### 3.6 Stage‐Dependent Transcriptional Remodeling of Cochlear Mitochondrial Quality‐Control–Related Programs Revealed by Targeted qPCR

#### 3.6.1 Energy‐Sensing and Autophagy‐Initiation Transcripts Show Non‐Linear Stage‐Dependent Remodeling

**FIGURE 6:**
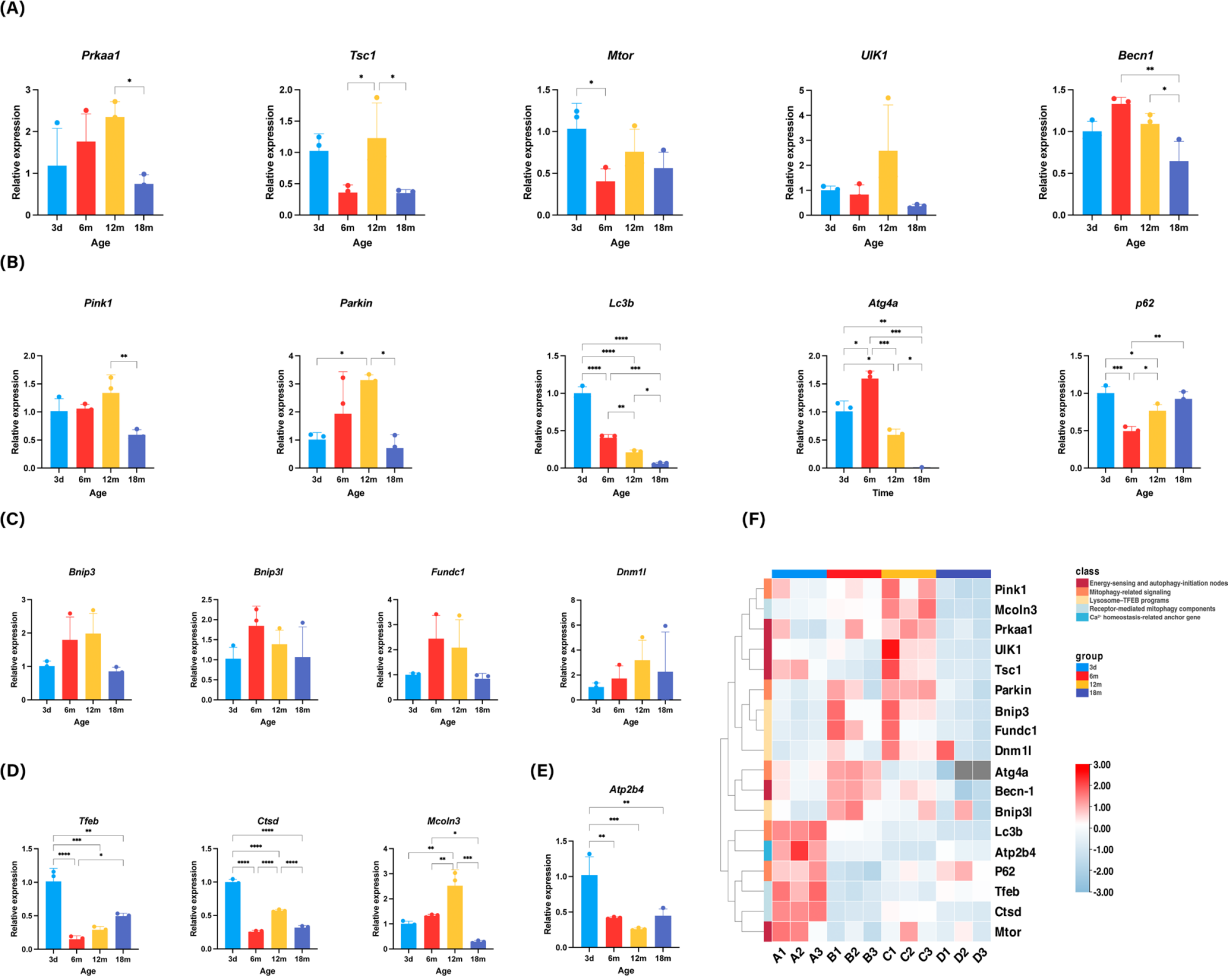
RT–qPCR reveals stage‐dependent transcriptional remodeling of cochlear mitophagy and autophagy–lysosome programs during aging. These qPCR results are presented as descriptive transcriptional changes and should not be interpreted as direct measurements of autophagic flux, mitophagy activity, or lysosomal function. mRNA expression of mitochondrial quality‐control–related genes was measured in cochlear tissues from SAMP8 mice at postnatal Day 3 (3d), 6 months (6 m), 12 months (12 m), and 18 months (18 m). Relative expression was calculated using the 2^−ΔΔCt method, normalized to β‐Actin and Gapdh, and visualized as log2 fold change. Genes are presented by functional modules: (A) Energy sensing and autophagy initiation/regulatory module (Prkaa1, Tsc1, Mtor, Ulk1, Becn1); (B) PINK1/Parkin axis and autophagy execution/cargo handling module (Pink1, Park2/Prkn, Lc3b, Atg4a, Sqstm1/p62); (C) Receptor‐mediated mitophagy and mitochondrial remodeling module (Bnip3, Bnip3l, Fundc1, Dnm1l); (D) TFEB‐associated lysosome/endosome module (Tfeb, Ctsd, Mcoln3); (E) Atp2b4 as a Ca^2+^ homeostasis–related candidate anchor gene; (F) Heatmap summarizing age‐dependent expression profiles of the above genes, with color indicating log2 fold change relative to the reference group (see color scale). Unless otherwise specified, plots show means with SD error bars; sample sizes and statistical units are summarized in Table S1. Significance is coded as *p* < 0.05 (*), < 0.01 (**), < 0.001 (***), < 0.0001 (****).

Targeted qPCR profiling of cochlear tissue across 3d, 6 m, 12 m, and 18 m revealed a non‐linear remodeling of upstream energy‐sensing and autophagy‐initiation regulators. Prkaa1 (AMPKα1) increased toward 12 m and then decreased at 18 m (12 m vs. 18 m, *p* < 0.05; Figure 6A), consistent with a transient activation of energy‐stress signaling. Tsc1 displayed a similar “dip–peak–drop” pattern, with lower levels at 6 m, a rebound at 12 m, and reduction again at 18 m (*p* < 0.05). MTOR was highest at 3d and remained reduced in adult/aged groups, with significant differences between early and later time points. Notably, Ulk1 exhibited a pronounced elevation at 12 m followed by marked suppression at 18 m, whereas Becn1 peaked at 6 m and progressively declined thereafter, reaching its lowest level at 18 m (*p* < 0.05). Together, these data indicate that cochlear autophagy initiation is not monotonically suppressed with age, but rather shows a mid‐life induction that is followed by late‐stage attenuation, consistent with non‐linear, stage‐dependent transcriptional remodeling across the sampled time points.

#### 3.6.2 Mitophagy‐Related Transcripts Suggest a Shift From Mid‐Life Induction Toward a Late‐Stage Burden‐Associated Signature

Mitophagy‐associated regulators further supported a staged remodeling. The PINK1–Parkin axis showed evidence of induction during mid‐life (Figure 6B): Pink1 increased at 12 m and declined at 18 m (*p* < 0.05), while Parkin was elevated at 6–12 m and reduced at 18 m (*p* < 0.05). In parallel, canonical autophagosome machinery‐related transcripts showed marked stage‐dependent changes: Lc3b decreased sharply from 3d to 6 m and remained profoundly suppressed through 12–18 m (*p* < 0.05). Atg4a displayed a transient elevation at 6 m followed by progressive reduction, reaching near‐minimal levels at 18 m (*p* < 0.05). Conversely, the cargo adaptor p62/SQSTM1 was reduced at 6 m but increased again with aging, approaching higher levels by 18 m (*p* < 0.05), a pattern that, when considered together with sustained Lc3b suppression, is consistent with a burden‐associated transcriptional signature but does not by itself establish impaired degradative flux.

#### 3.6.3 Receptor‐Mediated Mitophagy Components Show Mid‐Life Induction With Reduced Expression at Advanced Age; Mitochondrial Dynamics Trends Upward

Receptor‐mediated mitophagy components showed a broadly similar temporal structure. Bnip3, Bnip3l (NIX), and Fundc1 were elevated in adult/mid‐life groups (*p* < 0.05) and were lower at 18 m, consistent with diminished engagement of receptor‐mediated mitophagy pathways at advanced age. Dnm1l (mitochondrial fission‐related) exhibited an overall upward trend with age but with substantial variability, suggesting that mitochondrial remodeling dynamics may shift with aging even when transcriptional changes are heterogeneous (Figure 6C).

#### 3.6.4 Lysosome–TFEB Programs Are Suppressed Relative to Neonatal Levels and Exhibit Partial, Stage‐Specific Re‐Engagement

Given that mitophagy depends on lysosomal degradative capacity, we examined TFEB–lysosome‐associated transcripts. TFEB and the lysosomal protease Ctsd were highest at 3d and significantly reduced across later ages, with only partial recovery at 12–18 m. In contrast, the lysosomal/endolysosomal channel Mcoln3 showed a distinct pattern characterized by a prominent increase at 12 m followed by a marked decrease at 18 m (*p* < 0.05; Figure 6D), suggesting a stage‐specific engagement of lysosome‐linked signaling that does not persist into advanced age.

#### 3.6.5 ATP2B4 Expression Decreases From Neonatal to Adult Stages With Partial Late‐Age Rebound

Finally, Atp2b4—nominated as an aging‐associated anchor candidate from the adult‐versus‐aged hair‐cell dataset—showed higher expression at 3d, a reduction across 6–12 m, and a partial increase at 18 m (*p* < 0.05; Figure 6E). This divergence from the external adult‐versus‐aged nomination likely reflects differences in biological context (whole cochlea vs. hair‐cell–enriched transcriptomes) and the inclusion of a neonatal baseline, together supporting the interpretation of ATP2B4 as a context‐dependent stress/homeostasis node rather than a unidirectional aging marker.

To reduce potential developmental confounding by the 3d group, we additionally performed adult‐only sensitivity analyses restricted to the 6 m, 12 m, and 18 m groups. Recalculation of transcript‐level z‐scores and derived molecular indices using adult‐group samples only preserved the principal directionality of the key molecular signatures, including the relative late‐age increase in p62/Sqstm1, sustained suppression of Lc3b, and a corresponding shift in the adult‐only flux–burden index, together with a partial late‐age rebound of Atp2b4 (Figure S5).

### 3.7 Cross‐Layer Trajectory Alignment Reveals Divergence Between Autophagy Burden and TFEB–Lysosome Transcriptional Coupling During Aging

To integrate ultrastructural, synaptic, functional, and transcriptional readouts under incomplete cross‐assay matching, we conducted age‐point coupling plots using age‐group means (3d, 6 m, 12 m, 18 m; *n* = 3/4 age points). Because the 3d group represents a developmental stage, integrative plots spanning 3d–18 m are interpreted here as descriptive age‐point summaries across developmental and aging stages, whereas adult aging inferences are anchored primarily in the 6 m–18 m comparisons.

**FIGURE 7:**
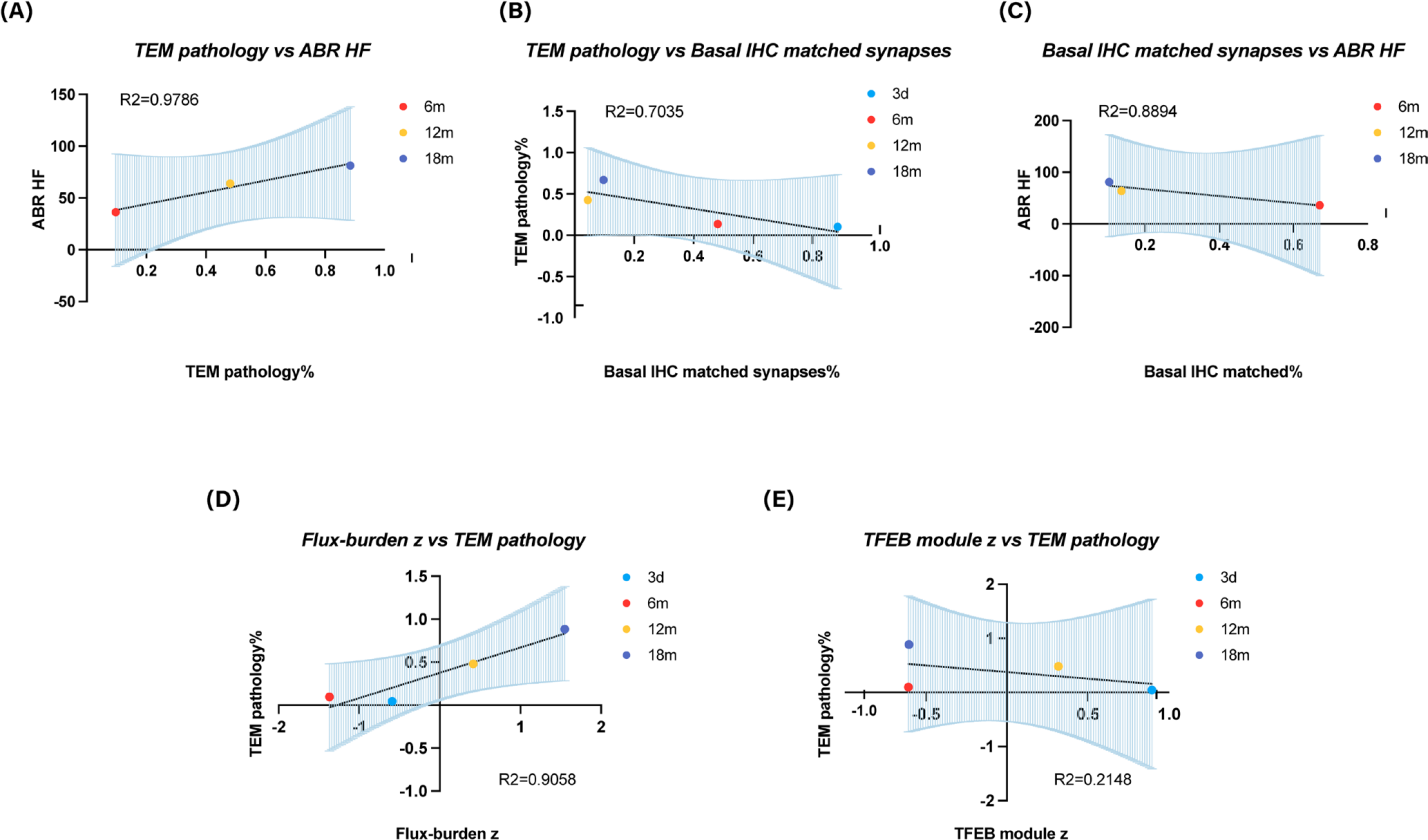
Descriptive age‐point coupling across ultrastructural, synaptic, functional, and molecular readouts reveals divergence between flux–burden and TFEB–lysosome alignment. (A) Age‐point coupling between TEM‐derived mitochondrial pathology burden and ABR high‐frequency threshold (HF (avg_24_32)). (B) Coupling between mitochondrial pathology burden and the basal‐turn IHC matched synapse metric. (C) Coupling between the basal‐turn IHC matched synapse metric and ABR HF. (D) Coupling between the flux–burden index (z(p62) − z(LC3B)) and mitochondrial pathology burden. (E) Coupling between the TFEB module z‐score (mean of z(Tfeb), z(Ctsd), z(Mcoln3)) and mitochondrial pathology burden. Linear fits and *R* ^2^ were used strictly as descriptive indices of trajectory alignment, not as inferential statistics.

Across aging, TEM‐derived mitochondrial pathology burden aligned tightly with high‐frequency auditory thresholds (ABR HF (avg_24_32)), exhibiting a near‐linear monotonic relationship across age points (*R* ^2^ = 0.9786; Figure 7A). This alignment is consistent with parallel age‐associated changes in mitochondrial ultrastructural burden and functional deterioration in the high‐frequency domain. In the same framework, mitochondrial pathology burden showed an inverse trajectory alignment with basal‐turn IHC matched synapse metric (*R* ^2^ = 0.7035; Figure 7B), indicating inverse trajectory alignment between basal cochlear synaptic coupling and mitochondrial pathology burden across age points. Consistently, the basal‐turn IHC matched synapse metric exhibited a strong inverse alignment with ABR HF_avg_24_32 (*R* ^2^ = 0.8894; Figure 7C), reinforcing a coherent cross‐layer trajectory linking basal synaptic uncoupling to high‐frequency threshold elevation.

At the transcriptional level, we next contrasted two descriptive transcriptional indices: a flux–burden signature versus a TFEB–lysosome module. The flux–burden index, defined as z(p62) − z(LC3B), showed a strong positive alignment with mitochondrial pathology burden (*R* ^2^ = 0.9058; Figure 7D), consistent with an age‐associated increase in autophagy/clearance “burden” accompanying damage accumulation. In contrast, the TFEB module z‐score (mean of z(Tfeb), z(Ctsd), z(Mcoln3)) showed only weak alignment with mitochondrial pathology burden (*R* ^2^ = 0.2148; Figure 7E), suggesting that lysosome biogenesis/lysosomal competency programs may be stage‐dependent, non‐linear, or temporally uncoupled from the monotonic accumulation of ultrastructural injury. Consistent with this interpretation, adult‐only descriptive plots based on the 6 m, 12 m, and 18 m group means preserved the same qualitative age‐ordered relationships among mitochondrial pathology burden, high‐frequency ABR loss, basal‐turn matched IHC synapse metrics, and the recalculated adult‐only transcriptional indices (Figure S6). These fits and their *R* ^2^ values are presented for descriptive visualization only.

Together, these descriptive coupling plots support a working model in which mitochondrial ultrastructural burden, basal synaptic uncoupling, and high‐frequency functional decline change in parallel with age, with stronger descriptive alignment to the flux–burden signature than to the TFEB–lysosome module (Figure S3).

## 4 Discussion

Age‐related sensory decline is increasingly recognized as a multi‐system disorder that compromises communication, mobility, and healthy aging (Montero‐Odasso et al. 2022; Michanski et al. 2023; Sun et al. 2023; Tighilet and Chabbert 2023). Yet most mechanistic work has focused on the cochlea, whereas vestibular aging—and how it aligns or diverges from cochlear vulnerability—remains less well resolved at integrated molecular–cellular–functional resolution (Tighilet and Chabbert 2023). The present study addressed this gap by examining whether aging in SAMP8 mice is accompanied by progressive mitochondrial injury together with remodeling of autophagy/mitophagy‐related transcriptional signatures, which in turn aligns with synaptic pathology and measurable auditory–vestibular functional decline. Across four‐age series, our data define a cross‐scale pattern in which (i) accumulation of mitochondrial ultrastructural pathology, (ii) coordinated shifts in autophagy/lysosome and mitophagy‐associated transcriptional modules (including a Ca^2+^‐homeostasis–anchored axis), (iii) region‐resolved synaptic degeneration, and (iv) deterioration of ABR and VsEP phenotypes (Wan et al. 2019; Liu et al. 2024) become increasingly evident with age.

A key observation is that functional decline unfolds across both auditory and vestibular systems but with partially distinct readouts, consistent with differential end‐organ vulnerability. ABR thresholds increased broadly across frequencies, with a prominent high‐frequency component—an archetypal presbycusis‐like phenotype that parallels the broader clinical burden of hearing loss and its downstream consequences. In contrast, vestibular impairment was captured most sensitively by VsEP threshold shifts and latency changes, whereas amplitude‐related metrics were comparatively preserved (Wan et al. 2019). This pattern aligns with the concept that aging often perturbs sensitivity and temporal fidelity before it uniformly depresses response magnitude, and it underscores the value of multiplexed vestibular waveform metrics rather than a single endpoint (Wan et al. 2019). From a translational perspective, the parallel progression of hearing and vestibular deficits is highly relevant because vestibular dysfunction contributes directly to falls and disability in older adults, and hearing loss is associated with cognitive and functional decline trajectories in epidemiological cohorts (Livingston et al. 2020; Croll et al. 2021; Montero‐Odasso et al. 2022; Tighilet and Chabbert 2023).

The anatomical substrate underlying these functional trajectories is increasingly framed as “synaptopathy‐first” vulnerability in the inner ear. Previous work established that cochlear synapses and afferent terminals can degenerate even when hair cells persist, producing enduring neural deficits that are not fully captured by threshold shifts alone (Liu et al. 2024). Age‐related cochlear synaptopathy has since been demonstrated as a progressive hallmark in murine models (Peineau et al. 2021; Liu et al. 2024). Our region‐stratified ribbon synapse analysis extends this framework in two ways. First, it strengthens the temporal and spatial linkage between synaptic pathology and functional readouts by aligning tonotopic synapse vulnerability with broadband ABR deterioration, particularly at the basal/high‐frequency end where metabolic and excitotoxic stress are often greatest (Peineau et al. 2021; Liu et al. 2024). Second, it places the vestibular system into the same mechanistic narrative: vestibular synaptic deficits and altered coupling of pre‐ and post‐synaptic markers are consistent with emerging evidence that vestibular synaptopathy contributes to age‐related otolith dysfunction (Wan et al. 2019; Michanski et al. 2023). Together, these findings support the view that aging‐related sensory decline is not explained solely by hair‐cell survival. Rather, cochlear aging was characterized by comparatively preserved hair‐cell numbers relative to the extent of synaptic and functional decline, despite region‐selective OHC loss, whereas vestibular aging involved more overt reductions in macular hair‐cell density and structural disorganization. This pattern highlights synaptic vulnerability and afferent connectivity as key components of sensory aging across both cochlear and vestibular end organs (Wan et al. 2019; Tighilet and Chabbert 2023; Liu et al. 2024), although the vestibular findings primarily extend this comparative framework at the physiological and synaptic levels and do not yet match the cochlear observations in mechanistic depth.

Neural remodeling provides an additional layer of vulnerability that can amplify synaptic loss. Although the cochlea and vestibular maculae differ in afferent organization and coding strategies, both depend on precisely patterned peripheral innervation. Our NF200‐based quantification in the cochlea indicates age‐associated remodeling of fiber‐associated area fraction in a region‐dependent manner. Rather than treating this solely as a secondary marker, it is useful to interpret it as a structural correlate of “connectivity reserve”: as synapses degenerate, peripheral fibers and terminals can retract, reorganize, or exhibit reduced presence within defined regions of interest, thereby compounding synaptic uncoupling (Liu et al. 2024). This interpretation is consistent with the broader framework of primary neural degeneration in the inner ear following insults that do not necessarily eliminate hair cells immediately. Our approach emphasizes spatially resolved quantification, which is essential because tonotopic regions can differ substantially in metabolic demand and susceptibility (Peineau et al. 2021; Liu et al. 2024).

At the ultrastructural level, our data provide an important morphological anchor for interpreting these degenerative changes. Aging increased the proportion of “pathological” mitochondria as defined by swelling/rounding, vacuolization, cristae disruption, and outer membrane rupture—features that collectively indicate impaired mitochondrial integrity and bioenergetic competence (Böttger and Schacht 2013; Oh et al. 2020). In line with established hallmarks of mitochondrial aging, we also observed electron‐lucent matrix, loss of cristae organization, and a shift toward more rounded mitochondria, which commonly reflect permeability changes, osmotic imbalance, and disrupted fission–fusion homeostasis (Böttger and Schacht 2013). These ultrastructural signatures align with prior work implicating mitochondrial respiratory chain dysfunction and oxidative stress as core contributors to cochlear aging and with genetic evidence that mitochondrial antioxidant capacity can modulate age‐related hearing loss trajectories (Böttger and Schacht 2013; Xiao et al. 2025). Extending to mitophagy specifically, age‐associated reductions in mitophagy capacity have been reported in the cochlea of aging mice (Oh et al. 2020), providing a plausible mechanistic bridge between accumulated mitochondrial pathology and subsequent cellular dysfunction.

Building on this ultrastructural foundation, our molecular results suggest that coordinated stage‐dependent remodeling of autophagy/lysosome‐ and mitophagy‐associated transcriptional modules rather than isolated single‐gene effects (Böttger and Schacht 2013; Oh et al. 2020; Chen et al. 2024; Guo et al. 2025). This module‐based framing is particularly relevant because autophagy competence depends not only on initiation (e.g., AMPK–ULK1, mTOR–TSC1) but also on execution and clearance capacity (lysosomal biogenesis, proteolysis, and ion homeostasis) (Chen et al. 2024). TFEB is widely regarded as a central transcriptional regulator linking lysosomal biogenesis with autophagic capacity (Chen et al. 2024; He et al. 2020, 2021), consistent with the protective role of Rictor/mTORC2 activation against sensorineural hearing loss (Fu et al. 2022). In parallel, lysosomal Ca^2+^ signaling can activate TFEB via calcineurin‐dependent pathways, thereby functionally coupling Ca^2+^ dynamics to degradative capacity (Chen et al. 2024). Within this framework, our transcriptomic nomination of Atp2b4 should be viewed as a hypothesis‐generating clue rather than mechanistic validation: Atp2b4 (PMCA4) may represent a candidate stress/homeostasis‐associated node for Ca^2+^ extrusion demand. Its association with lysosome/TFEB‐linked genes and autophagy‐related markers supports an interpretation in which aging imposes increasing Ca^2+^‐handling burden that is met by compensatory transcriptional shifts in degradative pathways. At the level of mitophagy, both PINK1/Parkin‐dependent ubiquitin signaling and receptor‐mediated pathways (e.g., FUNDC1, BNIP3/NIX) provide complementary routes for selective mitochondrial clearance (Böttger and Schacht 2013; Oh et al. 2020; Chen et al. 2024). The observed remodeling across these axes is therefore consistent with a system‐level attempt to maintain mitochondrial quality under accumulating stress—an attempt that ultimately becomes insufficient, as reflected by increased mitochondrial pathology and progressive synaptic/functional deficits (Böttger and Schacht 2013; Oh et al. 2020; Chen et al. 2024).

A major strength of this study is its integrative evidence chain across modalities despite incomplete one‐to‐one pairing at the individual‐animal level. By leveraging age‐group means for coupling plots, we tested whether molecular indices (e.g., TFEB–lysosome module score; flux–burden index) align with trajectories of mitochondrial pathology, synaptic readouts, and function. These coupling analyses are best interpreted as descriptive trajectory alignment rather than inferential correlation: they support coherence of an age‐aligned cross‐scale pattern at the level of aging progression but do not establish causality. Nonetheless, the fact that independent assays—TEM ultrastructure, regional synapse quantification, nerve fiber metrics, ABR, and VsEP—converge on a consistent directionality strengthens the biological interpretation that mitochondrial injury and quality‐control remodeling are closely associated with connectivity loss and sensory dysfunction, while their causal ordering remains unresolved (Böttger and Schacht 2013; Wan et al. 2019; Oh et al. 2020; Tighilet and Chabbert 2023; Chen et al. 2024; Liu et al. 2024). This addresses a common critique in aging studies, namely that any single assay can be ambiguous, but convergent cross‐level patterns are harder to dismiss as incidental.

From a clinical perspective, the cochlea–vestibule comparison is more than an anatomical contrast: it clarifies why older adults frequently experience combined hearing difficulty and balance impairment, a syndromic constellation strongly linked to falls, reduced independence, and broader health risks (Montero‐Odasso et al. 2022; Tighilet and Chabbert 2023). In addition, hearing loss has been linked to incident cognitive decline and dementia risk in population studies (Livingston et al. 2020; Croll et al. 2021; Liang et al. 2021), reinforcing the relevance of mechanistic pathways that could be therapeutically leveraged (Livingston et al. 2020; Liang et al. 2021). Our data motivate the hypothesis that strategies aimed at sustaining mitochondrial quality control (mitophagy competence, lysosomal capacity, and Ca^2+^ homeostasis) may be relevant to both auditory and vestibular aging trajectories, although this possibility requires direct functional testing. (Böttger and Schacht 2013; Oh et al. 2020; Tighilet and Chabbert 2023; Chen et al. 2024). Importantly, our findings also emphasize that “early targets” may reside at the level of synapses and connectivity, suggesting that interventions introduced before overt hair cell loss may yield disproportionate functional gains (Peineau et al. 2021; Liu et al. 2024).

Several limitations should be considered when interpreting these findings. First, the cross‐endpoint integrative analyses were based primarily on age‐group means because complete individual‐level multimodal pairing was not available for all assays; these plots should therefore be interpreted as descriptive and hypothesis‐generating. Second, the qPCR analyses were performed on whole‐cochlea tissue and capture transcript‐level remodeling only; they do not provide direct measurements of protein abundance, autophagic flux, mitophagy activity, or lysosomal function. Third, the 3d group represents a developmental rather than young‐adult stage, so trajectories spanning 3d to adulthood likely reflect maturation in addition to aging; adult aging inferences are therefore best anchored in the 6–18 m comparisons. Fourth, TEM scoring relied on a predefined binary morphology rubric and a limited sample size, which supports directional ultrastructural observations but not strong mechanistic inference. Fifth, cochlear and vestibular evidence depth was asymmetric, with deeper ultrastructural and molecular resolution on the cochlear side than on the vestibular side. Finally, transcriptome‐nominated candidates such as Atp2b4 remain unvalidated within the present dataset and should be considered priorities for future perturbation‐based studies. In addition, although SAMP8 is a well‐established accelerated aging model, generalization to other strains and to human aging requires careful validation; future work should incorporate comparative models and mechanistic perturbations (Tighilet and Chabbert 2023; Liu et al. 2024).

Looking forward, several directions can strengthen causal inference and translational impact. Spatially resolved or single‐cell transcriptomics could localize the Atp2b4–Ca^2+^/lysosome signal and define which cell populations drive module shifts (Sun et al. 2025). Protein‐level validation and functional assays of lysosomal capacity and mitophagy competence would help distinguish transcriptional induction from effective flux. Longitudinal designs or interventions targeting mitochondrial dynamics and lysosomal biogenesis could test whether preserving quality control attenuates synaptopathy and stabilizes ABR/VsEP trajectories (Böttger and Schacht 2013; Oh et al. 2020; Chen et al. 2024).

## 5 Conclusion

In SAMP8 mice, aging was accompanied by coordinated cochlear and vestibular functional decline together with region‐resolved synaptic vulnerability, cochlear fiber remodeling, and increasing mitochondrial ultrastructural abnormalities. Across an age series, rising ABR and VsEP thresholds—together with vestibular latency changes—co‐occurred with region‐resolved synaptic vulnerability and cochlear fiber remodeling, while transmission electron microscopy revealed an increasing burden of pathological mitochondria consistent with progressive organelle injury. Guided by adult‐versus‐aged transcriptomic nomination, targeted qPCR further indicated remodeling of autophagy/mitophagy–lysosome programs and highlighted Atp2b4 as a Ca^2+^‐handling–associated candidate anchor. Collectively, these findings support age‐aligned associations among mitochondrial injury, molecular remodeling, synaptic vulnerability, and auditory–vestibular dysfunction. The study provides an integrated comparative framework and a set of testable hypotheses for future work aimed at validating flux‐level mechanisms and evaluating pathway‐targeted strategies to preserve hearing and balance with aging.

## Author Contributions

**Jingyi Xie:** conceptualization, formal analysis, investigation, data curation, software, visualization, writing – original draft. **Xujia Zhang:** software, validation, formal analysis, data curation. **Jinyi Tian:** software, investigation, validation, methodology. **Yulu Yan:** software, validation, data curation. **Ke Shi:** validation, data curation. **Yongqi Pan:** validation, data curation. **Yanni Zhang:** methodology, resources. **Zichen Chen:** methodology. **Jianbin Sun:** resources. **Hui Lv:** project administration. **Jingguo Chen:** project administration. **Xiaoyong Ren:** supervision, project administration. **Teru Kamogashira:** conceptualization, methodology. **Xiaotong Zhang** (Corresponding author): supervision, project administration, writing – review and editing. **Ying Gao** (Corresponding author): conceptualization, methodology, software, validation, resources, visualization, supervision, funding acquisition, writing – review and editing.

## Funding

This work was supported by the National Natural Science Foundation of China [grant number 82101224]; the Institute Foundation's Free Exploration Project of the Second Affiliated Hospital of Xi'an Jiaotong University [grant number 2020YJ(ZYTS)043]; the Xi'an Jiaotong University Education Foundation [grant number XJYG2025‐SFJJ2032]; IIT Clinical Research Fund of The Second Affiliated Hospital of Xi'an Jiaotong University [grant number: M020].

## Conflicts of Interest

The authors declare no conflicts of interest.

## Supporting information


**Figure S1:** Global immunofluorescence map of hair cells in the saccule and utricle of different ages.
**Figure S2:** Thematic_DEG_Summary.
**Figure S3:** Mechanism Diagram.
**Figure S4:** Technical Roadmap.
**Figure. S5**. Adult‐only sensitivity analysis of key cochlear transcriptional signatures and derived molecular indices. Adult‐only analyses were restricted to the 6 m, 12 m, and 18 m groups to reduce developmental confounding from the 3d cohort. (A) Adult‐only Lc3b expression. (B) Adult‐only p62/Sqstm1 expression. (C) Adult‐only Atp2b4 expression. (D) Adult‐only flux–burden index recalculated from adult‐group data only, defined as z(p62) − z(Lc3b). (E) Adult‐only TFEB–lysosome module recalculated from adult‐group data only, defined as mean[z(Tfeb), z(Ctsd), z(Mcoln3)]. *z*‐scores and derived composite indices were recalculated using only adult‐group samples. These results are presented as descriptive transcriptional summaries and should not be interpreted as direct functional measurements of autophagic flux, mitophagy activity, or lysosomal function.
**Figure S6:** Adult‐only descriptive integrative plots across mitochondrial ultrastructure, synaptic integrity, auditory function, and transcriptional indices. Adult‐only descriptive plots were generated using only the 6 m, 12 m, and 18 m group means. (A) Pathological mitochondria burden versus HF_avg_24_32. (B) Pathological mitochondria burden versus basal‐turn matched IHC synapse metric. (C) Basal‐turn matched IHC synapse metric versus HF_avg_24_32. (D) Adult‐only flux–burden index versus pathological mitochondria burden. (E) Adult‐only TFEB–lysosome module versus pathological mitochondria burden. Linear fits and R^2^ values are shown as descriptive indices of trajectory alignment only and should not be interpreted as inferential correlation statistics.

**Table S1:** Sample sizes and units of analysis.

**Table S2:** Correlation analysis about XY_GroupMeans.

**Table S3:** qPCR data (−log2).

## Data Availability

All relevant data are within the manuscript and its Additional files.
