# Supplementary material for: Comparative Cochlear–Vestibular Aging Reveals Age‐Aligned Mitochondrial Ultrastructural Burden, Mitophagy–Autophagy Remodeling, Synaptic Uncoupling, and Sensory Functional Decline
Source: Aging Cell. 2026 Jun 23;25(7):e70593. doi: 10.1111/acel.70593 (PMC13288170; doi:10.1111/acel.70593)
Supplement: Supplementary file 1 — Figure S1: Global immunofluorescence map of hair cells in the saccule and utricle of different ages. Figure S2: Thematic_DEG_Summary. Figure S3: Mechanism Diagram. Figure S4: Technical Roadmap. Figure. S5. Adult‐only sensitivity analysis of key cochlear transcriptional signatures and derived molecular indices. Adult‐only analyses were restricted to the 6 m, 12 m, and 18 m groups to reduce developmental confounding from the 3d cohort. (A) Adult‐only Lc3b expression. (B) Adult‐only p62/Sqstm1 expression. (C) Adult‐only Atp2b4 expression. (D) Adult‐only flux–burden index recalculated from adult‐group data only, defined as z(p62) − z(Lc3b). (E) Adult‐only TFEB–lysosome module recalculated from adult‐group data only, defined as mean[z(Tfeb), z(Ctsd), z(Mcoln3)]. z‐scores and derived composite indices were recalculated using only adult‐group samples. These results are presented as descriptive transcriptional summaries and should not be interpreted as direct functional measurements of autophagic flux, mitophagy activity, or lysosomal function. Figure S6: Adult‐only descriptive integrative plots across mitochondrial ultrastructure, synaptic integrity, auditory function, and transcriptional indices. Adult‐only descriptive plots were generated using only the 6 m, 12 m, and 18 m group means. (A) Pathological mitochondria burden versus HF_avg_24_32. (B) Pathological mitochondria burden versus basal‐turn matched IHC synapse metric. (C) Basal‐turn matched IHC synapse metric versus HF_avg_24_32. (D) Adult‐only flux–burden index versus pathological mitochondria burden. (E) Adult‐only TFEB–lysosome module versus pathological mitochondria burden. Linear fits and R2 values are shown as descriptive indices of trajectory alignment only and should not be interpreted as inferential correlation statistics. [file ACEL-25-e70593-s003.docx]

**Appendix A. Supplementary material**

Supplementary Fig. S1. Global immunofluorescence map of hair cells in the saccule and utricle of different ages

**
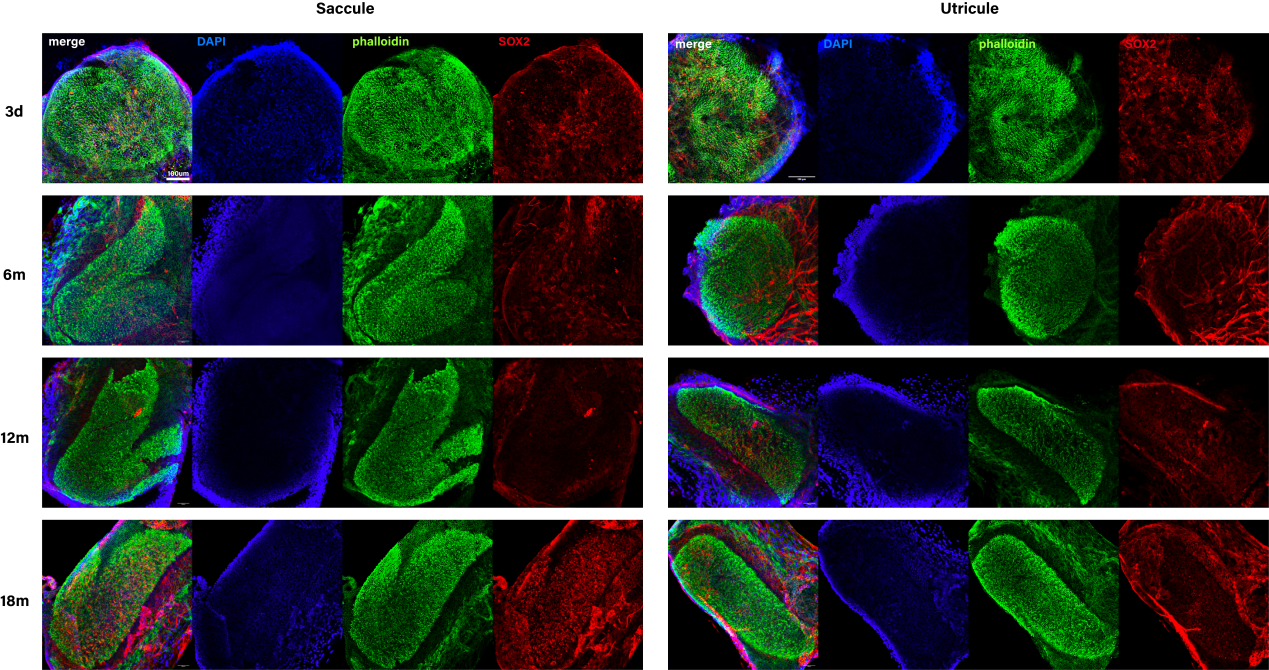
**

Supplementary Fig. S2. Thematic_DEG_Summary.


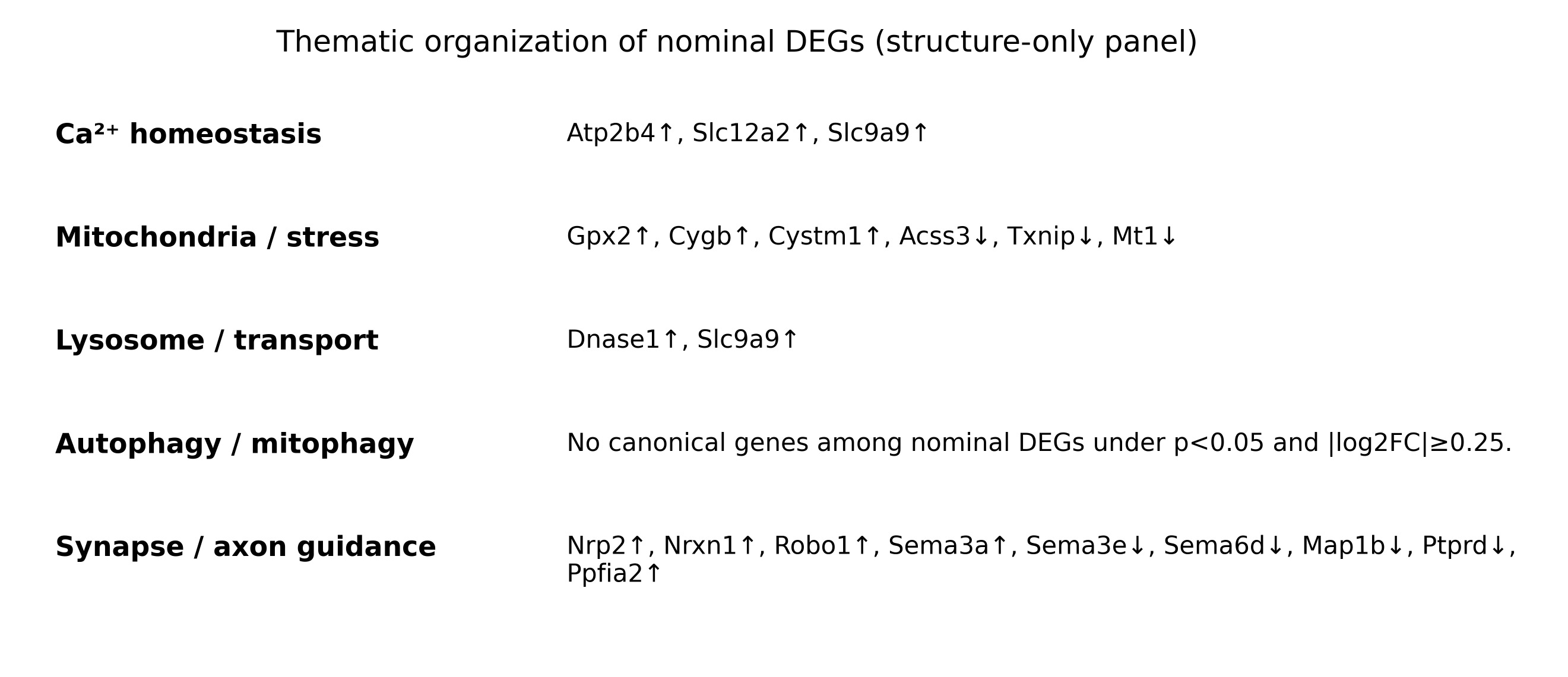


Supplementary Fig. S3. Mechanism Diagram


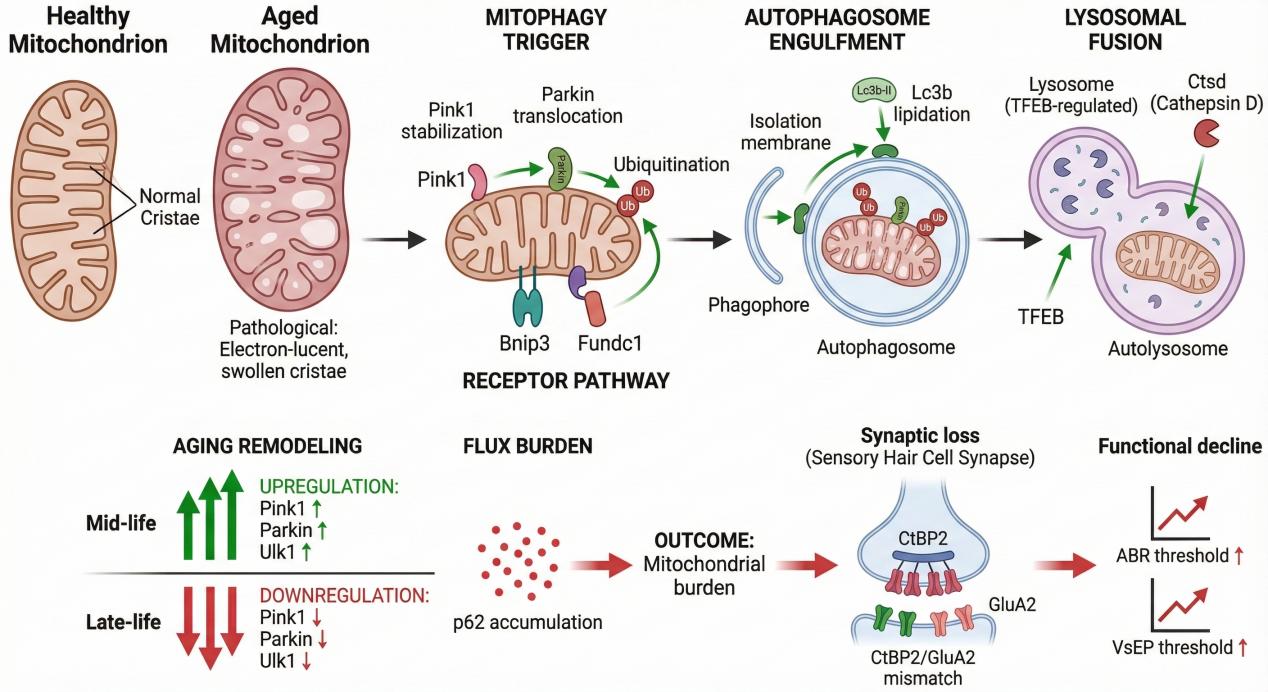


Supplementary Fig. S4. Technical Roadmap.


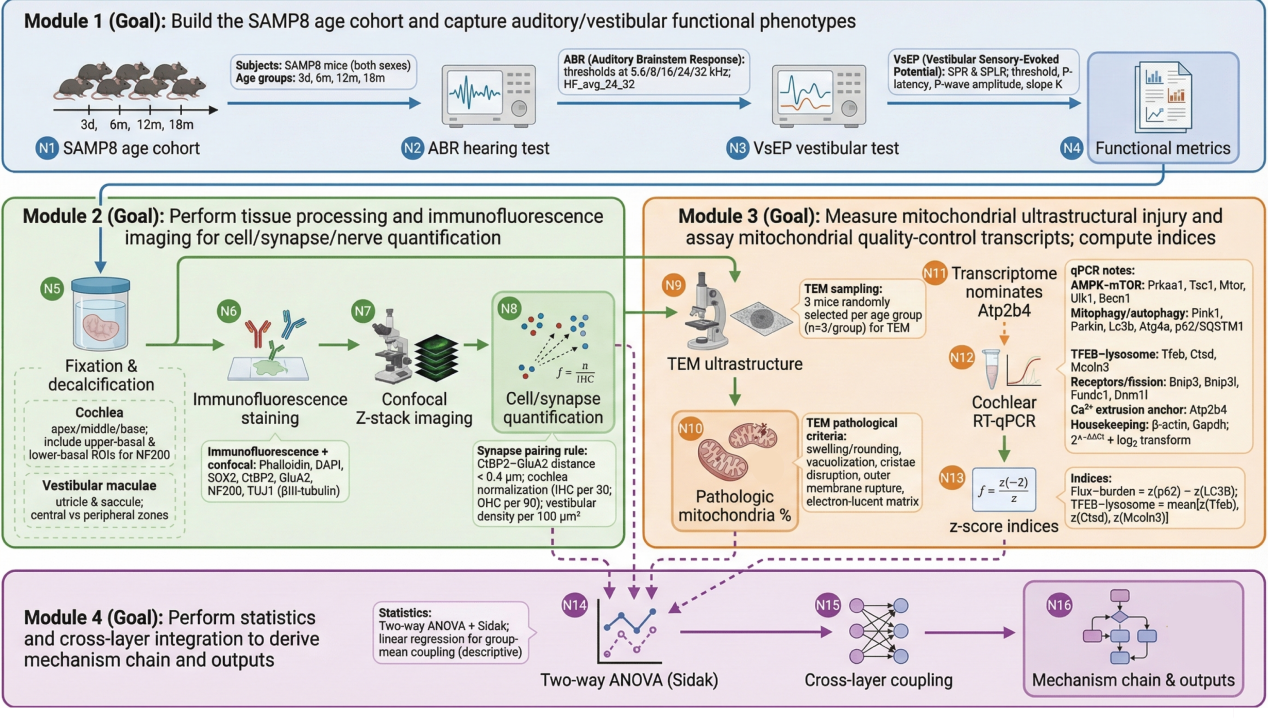


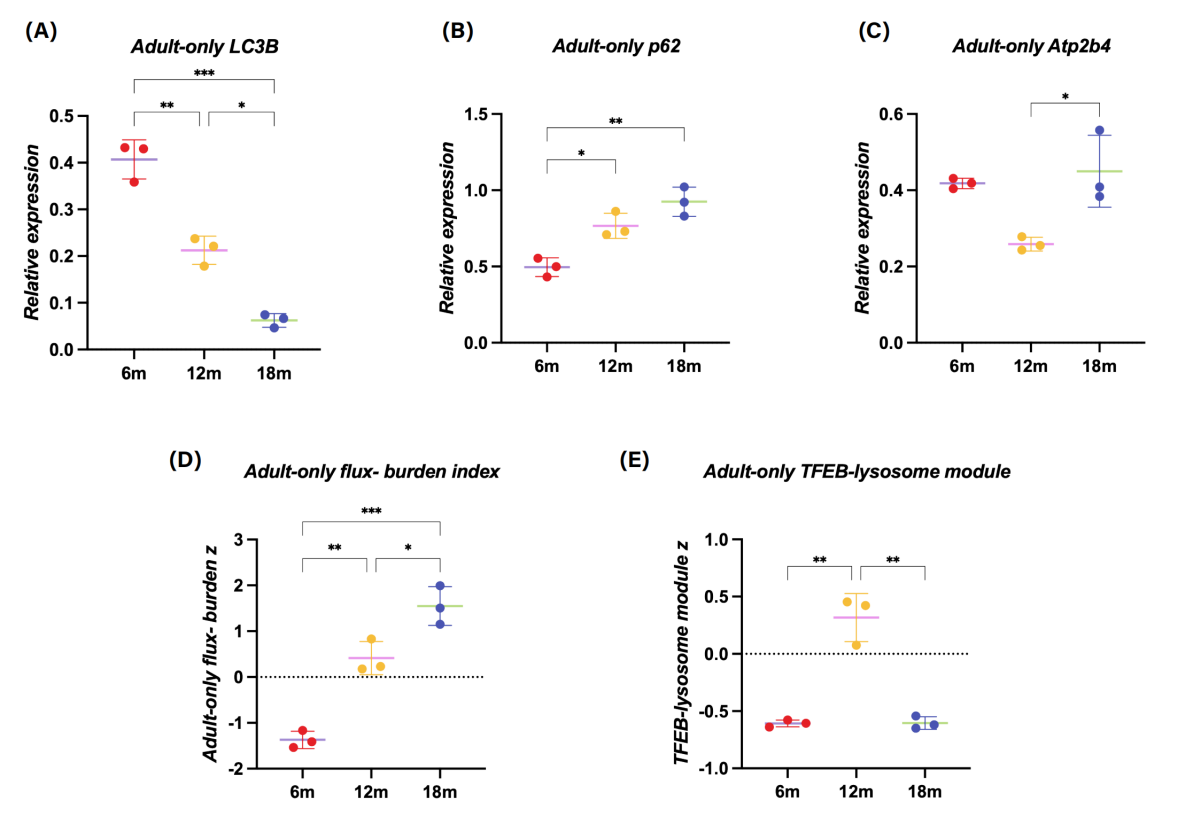


Supplementary Figure. S5. Adult-only sensitivity analysis of key cochlear transcriptional signatures and derived molecular indices. Adult-only analyses were restricted to the 6m, 12m, and 18m groups to reduce developmental confounding from the 3d cohort. (A) Adult-only Lc3b expression. (B) Adult-only p62/Sqstm1 expression. (C) Adult-only Atp2b4 expression. (D) Adult-only flux–burden index recalculated from adult-group data only, defined as z(p62) − z(Lc3b). (E) Adult-only TFEB–lysosome module recalculated from adult-group data only, defined as mean[z(Tfeb), z(Ctsd), z(Mcoln3)]. z-scores and derived composite indices were recalculated using only adult-group samples. These results are presented as descriptive transcriptional summaries and should not be interpreted as direct functional measurements of autophagic flux, mitophagy activity, or lysosomal function.


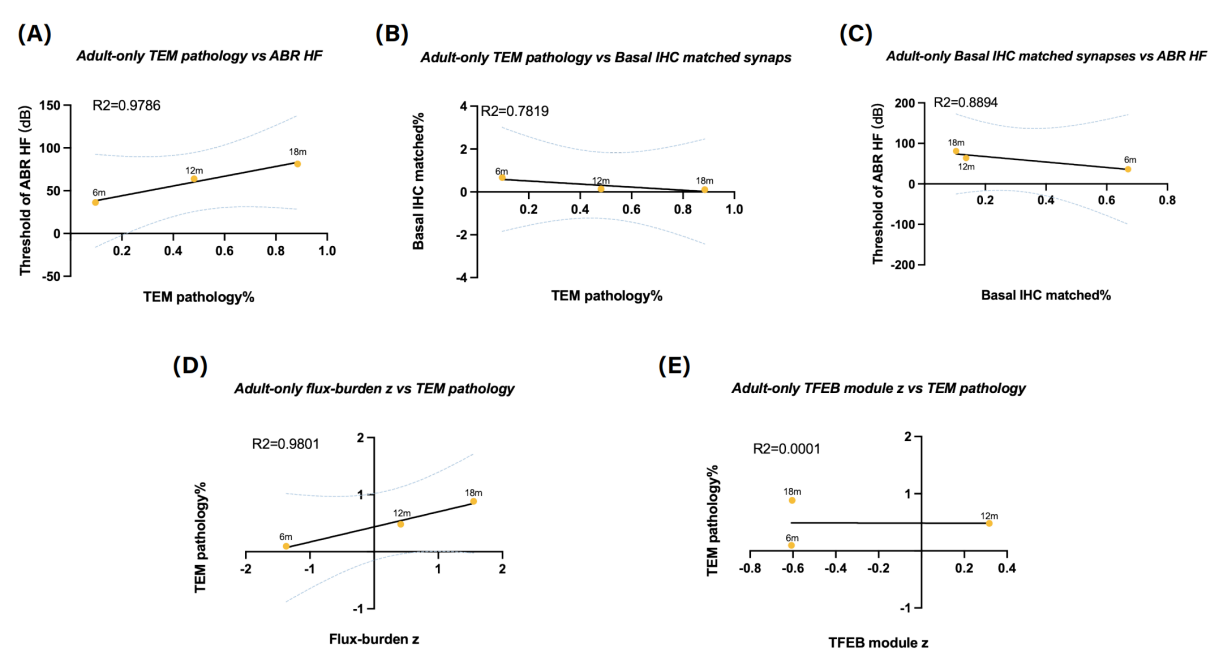


Supplementary Figure S6. Adult-only descriptive integrative plots across mitochondrial ultrastructure, synaptic integrity, auditory function, and transcriptional indices. Adult-only descriptive plots were generated using only the 6m, 12m, and 18m group means. (A) Pathological mitochondria burden versus HF_avg_24_32. (B) Pathological mitochondria burden versus basal-turn matched IHC synapse metric. (C) Basal-turn matched IHC synapse metric versus HF_avg_24_32. (D) Adult-only flux–burden index versus pathological mitochondria burden. (E) Adult-only TFEB–lysosome module versus pathological mitochondria burden. Linear fits and R² values are shown as descriptive indices of trajectory alignment only and should not be interpreted as inferential correlation statistics.

Supplementary Table. S1. Sample sizes and units of analysis.

Supplementary Table. S2. correlation analysis about XY_GroupMeans.

Supplementary Table. S3. qPCR data (-log2).
